# Robust intervention for oxidative stress-induced injury in periodontitis *via* controllably released nanoparticles that regulate the ROS-PINK1-Parkin pathway

**DOI:** 10.3389/fbioe.2022.1081977

**Published:** 2022-12-15

**Authors:** Xincong Li, Yue Zhao, Haoran Peng, Deao Gu, Chao Liu, Shuangshuang Ren, Leiying Miao

**Affiliations:** ^1^ Department of Cariology and Endodontics, Nanjing Stomatological Hospital, Medical School of Nanjing University, Nanjing, Jiangsu, China; ^2^ Department of Orthodontics, Nanjing Stomatological Hospital, Medical School of Nanjing University, Nanjing, Jiangsu, China

**Keywords:** periodontitis, oxidative stress injury, responsive controlled release, periodontal ligament stem cells (PDLSC), mitophagy

## Abstract

Oxidative stress in periodontitis has emerged as one of the greatest barriers to periodontal tissue restoration. In this study, we synthesized controlled drug release nanoparticles (MitoQ@PssL NPs) by encasing mitoquinone (MitoQ; an autophagy enhancer) into tailor-made reactive oxygen species (ROS)-cleavable amphiphilic polymer nanoparticles (PssL NPs) to regulate the periodontitis microenvironment. Once exposed to reactive oxygen species, which were substantially overproduced under oxidative stress conditions, the ROS-cleavable PssL was disintegrated, promoting the release of the encapsulated MitoQ. The released mitoquinone efficiently induced mitophagy through the PINK1-Parkin pathway and successfully reduced oxidative stress by decreasing the amount of reactive oxygen species. With the gradual decrease in the reactive oxygen species level, which was insufficient to disintegrate PssL, the release of mitoquinone was reduced and eventually eliminated, which contributed to a redox homeostasis condition and facilitated the regeneration of periodontal tissue. MitoQ@PssL NPs have great potential in the treatment of periodontitis *via* microenvironment-controlled drug release, which will provide a new avenue for periodontal regeneration and diseases related to imbalanced redox metabolism.

## Graphical Abstract

**Figure:**
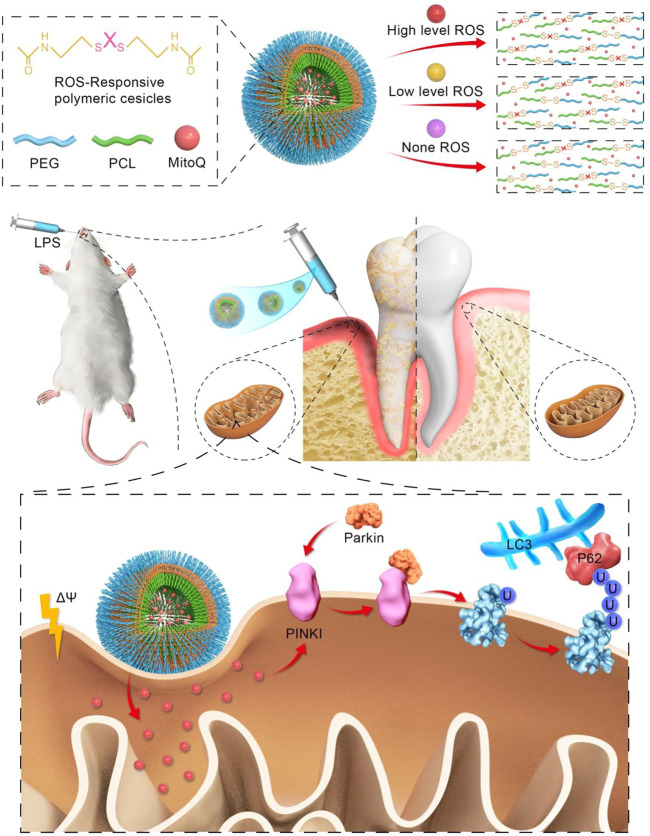
We introduce novel mitophagy enhancer, MitoQ@PssL NPs exert excellently anti-inflammatory effects by activating mitophagy and clearing partial mitochondrial ROS, thereby accelarating osteogenesis in periodontitis.

## 1 Introduction

Periodontitis, an inflammatory disease that affects the tissues supporting the teeth, is a common disease worldwide (Slots, 2017; Li et al., 2019). It leads to soft and hard tissue destruction, including the gingiva, periodontal ligament, cementum and alveolar bone (Sá et al., 2000). Currently, the clinical treatments for periodontitis include basic treatment, surgical treatment, and adjuvant drug treatment (Deas et al., 2016; Rajeshwari et al., 2019; Prietto et al., 2020). All these treatments provide inefficient periodontal tissue repair. Oxidative stress, which is characterized by the excess production of reactive oxygen species (ROS) that exceeds the threshold of tissue metabolism, is one of the main reasons for inefficient repair of periodontitis by those clinical treatments (Shi et al., 2021). Some antioxidants have been leveraged to eliminate oxidative stress and the corresponding excess ROS levels (Jiang, 2014; He et al., 2017). However, in some sense, the direct leverage of antioxidants rarely fundamentally eliminates excess ROS if these treatments do not activate some special pathways (Kanzaki et al., 2017). Some reports have shown that oxidative stress and corresponding excess ROS levels usually evoke autophagy-associated pathways, which in turn modestly mitigate oxidative stress and decrease ROS levels (Liu et al., 2017; Gao, 2019; Greabu et al., 2020). However, the levels of the self-activated autophagy pathways are typically low, which will not revolutionize the current landscape. Therefore, the introduction of some autophagy enhancers may be an important solution.

Currently, the commonly used autophagy enhancers are rapamycin and epigallocatechin gallate (EGCG) (Yun et al., 2020; Yao et al., 2021), which reduce oxidative stress and corresponding excess ROS levels. Despite their great potential, many drugs that activate or inhibit autophagy lack specificity (Ma et al., 2020). The specificity of some targets is not accurate; for example, 3-mA and wortmannin are nonselective PI3K inhibitors that inhibit a variety of PI3K family members (Kim et al., 2018). Similarly, although rapamycin selectively inhibits mTORC1, long-term administration will also promote the decomposition of mTORC2 (Sulkshane et al., 2021). Therefore, the target specificity of autophagy regulators plays a very important role in clinical development and application. In the study of regulators of mitochondrial autophagy (mitophagy), researchers have obtained an effective inhibitor (mdivi-1, a compound that inhibits autophagy by regulating mitochondrial morphological changes) (Nhu et al., 2021). The primary requirement for the development of mitophagy activators is that drugs smoothly pass through the mitochondrial membrane and act on mitochondria (Lampert et al., 2019), suggesting that we must modify drugs to help deliver them to mitochondria. Lipophilic TPP cations easily pass-through phospholipid bilayers because their charge is effectively dissipated and surrounded by a protective organic chemical array, enabling their accumulation in the mitochondrial matrix in response to the mitochondrial membrane potential (James et al., 2007). The TPP moiety on mitoquinone (MitoQ) enables its accumulation within mitochondria driven by the membrane potential. Therefore, MitoQ may be a potential regulator of mitophagy, but it has not been explored as a treatment for periodontitis. Excess ROS produced by mitochondria are a key mechanism of age-related vascular dysfunction. Matthew J. Rossman et al. (Pak et al., 2018) confirmed that MitoQ, an antioxidant targeting mitochondria, improves the function of vascular endothelial cells by reducing ROS produced by mitochondria and reducing atherosclerosis in aged mice. The findings from humans are consistent with previous preclinical observations, suggesting that MitoQ may be a promising treatment for age-related vascular dysfunction. MitoQ also reduces renal damage caused by ischemia‒reperfusion injury in rodent kidneys (Yu et al., 2020) and improves Alzheimer’s nerve injury (Yang et al., 2020). To a certain extent, mitophagy reverses the mitochondrial dysfunction caused by stem cell ageing (Fan et al., 2019). PINK1/Parkin-dependent mitophagy is a well-known posttranslational signaling cascade that recognizes the cargo through the polyubiquitination of mitochondrial proteins and the recruitment of the autophagy machinery (Xiao et al., 2017; Yao et al., 2019a; Quinn et al., 2020). However, MitoQ is only used as an oral medicine, mainly because of its limitations, including poor water dispersion, low stability, a short duration of action and a low unit effective concentration (Xing et al., 2017; Bao et al., 2018), resulting in its limited application in local periodontal tissues. Meanwhile, as most types of autophagy enhancers are small-molecule drugs, they face many limitations and challenges. First, these small-molecule drugs will be quickly eliminated from periodontal tissue, causing ineffective targeted autophagy. Second, blindly increasing the dose of small-molecule drugs administered to maintain a sufficient drug concentration in periodontal tissue will inevitably result in systemic toxicity (Wang et al., 2021; Ozbay et al., 2022).

Therefore, a superior treatment for periodontitis may be to introduce a drug delivery system for the targeted delivery of MitoQ. Compared with traditional periodontal local drug delivery systems (such as aerosols (Zhang et al., 2018), encapsulated cell capsules (Kim et al., 2021a), and gelatin (Chen et al., 2020), nanoparticle drug delivery systems have special advantages in terms of their biopharmaceutical and pharmacokinetic characteristics, including responsive controlled release drugs, long-term maintenance of the drug concentration, biodegradability and biocompatibility. For drug-releasing materials, practical clinical applications require drug carriers to achieve “zero release” and effective release of drugs after reaching the lesion. Polymer micelles are novel drug delivery systems formed from amphiphilic polymers by self-assembly. Amphiphilic polymers self-assemble into supermolecular structures (polymer micelles) by balancing hydrophilic and hydrophobic interactions. For the special high-level ROS microenvironment of periodontitis, we formed ROS-sensitive dynamic covalent bonds and ingeniously transformed the nanoparticle itself into an intelligent “gated switch” to ultimately obtain a ROS-sensitive and “self-gated” nanoparticle drug delivery system at the site of periodontitis. Here, we designed a nanoparticle that is capable of manipulating the responsive and controlled release of the autophagy enhancer MitoQ in the periodontal inflammatory microenvironment and then eliminating oxidative stress-induced injury through the activation of an autophagy-associated pathway, which is very important and represents an innovative and correct application of MitoQ as a targeted drug in periodontitis with safety and efficiency.

## 2 Materials and methods

### 2.1 Synthesis of MitoQ@PssL NPs

MitoQ@PssL NPs were prepared by an emulsion method following previous report (Qiu et al., 2021). Briefly, PssL (50 mg) was dissolved in deionized water (50 ml) while MitoQ (5 mg) was dissolved in dichloromethane (5 ml). After the dissolution, they were mixed and reacted under the ultrasonic treatment for about 30 min. Subsequently, MitoQ@PssL NPs were collected and purified by thrice centrifugation and washing with deionized water.

### 2.2 Characterization of PssL NPs and MitoQ@PssL NPs

The particle size and size distribution of PssL and MitoQ@PssL NPs in complete cell culture medium were assessed by dynamic light scattering (Brookhaven Instruments Co., United States). The morphologies of PssL and MitoQ@PssL NPs were examined using transmission electron microscopy (JEOL, Japan). Gel permeation chromatography (GPC) was used to measure the molecular weight and polydispersity of PssL. The number-average molecular weights (Mn), weight-average molecular weights (Mw), and polydispersity index (PDI, Mw/Mn) were determined using polystyrene as the reference.

### 2.3 Cell culture

Human periodontal ligament stem cells (hPDLSCs) were isolated from human periodontal ligament tissue, were purchased from Procell Life Science and Technology Co., Ltd. (Cat No: CP-H234, Procell, China). These cells were both cultured in Dulbecco’s modified Eagle’s medium (DMEM) (Keygen, China) containing 10% fetal bovine serum (FBS) (Gibco, United States) and 1% penicillin/streptomycin (HyClone, United States) at 37°C in 5% CO2. hPDLSCs between passages 3 and 6 were used for subsequent experiments. For osteogenic induction, the medium was changed to an osteogenic differentiation medium (Sigma, United States).

### 2.4 Measurement of intracellular and mitochondrial ROS

hPDLSCs were detached by trypsin and recultured on glass-bottom culture dishes with a density of 5 × 104 cells per dish. When hPDLSCs were reached near 80%, the lipopolysaccharide (LPS) was added to the medium at 8 μg/L for 2 h, then 200 ng/ml MitoQ@PssL NPs were added in the medium and cultured for 24 h. The intracellular level of ROS was measured by using a DCFH-DA probe (Keygen, China). The mitochondrial level of superoxide was measured by using the MitoSOXTM Red probe (Yeasen, China). The fluorescence of DCF and MitoSOX were observed at 488 nm and 510 nm separately under a laser scanning confocol microscope (LSCM, A1R, Nikon, Japan) and FACS flow cytometry (BD Biosciences, United States). Using the ImageJ program (Media Cybernetics, United States), the quantitative study of fluorescence intensity was carried out.

### 2.5 Efficacy detection of nanoparticles on mitochondrion structure and morphology

hPDLSCs were seeded onto glass-bottom cell culture dishes to ensure approximately 80% confluency and cultured with a medium containing LPS (8 μg/L) for 2 h. The cells were then treated with a complete medium containing MitoQ and MitoQ@PssL NPs (200 ng/ml) for 24 h and then treated with MitoTracker red FM probe (Yeasen, China) and observed under LSCM.

### 2.6 Assessment of *in-vitro* mitophagy expression

hPDLSCs were cultured in 24-well plates, and cultured with LPS and NPs as before. RT-PCR was used to evaluate the gene expression levels of mitophagy-related markers, including LC3Ⅰ, LC3Ⅱ, p62, PINK1, and Parkin. The primer sequences were listed in Supplementary Table S1. WB was used to evaluate the expression of mitophagy-related proteins. GAPDH was used as an internal control and antibodies-including LC3Ⅰ, LC3Ⅱ, p62, PINK1, and Parkin were purchased from Abcam.

hPDLSCs were seeded onto glass-bottom cell culture dishes and cultured with LPS and NPs as before. Mitochondria were stained by MitoTracker™ Red probe (Thermo Fisher Scientific, United States), and the colocalization of the mitochondrial of LC3, p62, PINK1, and Parkin were examed *via* LSCM. The structure and morphology of mitochondria were examined *via* TEM (Tecnai G2 Spirit Biotwin, United States).

### 2.7 Assessment of *in-vitro* osteogenic differentiation

hPDLSCs were cultured in 24-well plates with osteogenic induction medium. On day 7, real-time quantitative PCR (RT-PCR) was used to evaluate the gene expression levels of osteogenesis-related markers, including bone sialoprotein (BSP), type I collagen (COL-1), Runt-related transcription factor 2 (Runx2), osteocalcin (OCN), and osteopontin (OPN). The primer sequences were listed in Table S1 On day 7, ALP activity assay and ALP staining kits (Keygen, China) were used to analyze ALP activity. For calcium node staining at day 14, Alizarin Red S (Sigma, United States) was used. On day 14, WB was used to assess the expression of osteogenic-related proteins. GAPDH was used as an internal control and antibodies-including LC3Ⅰ, LC3Ⅱ, p62, PINK1, and Parkin were purchased from Sigma.

### 2.8 *In vivo* treatment of MitoQ@PssL NPs in the periodontitis disease model

Twenty-four Sprague-Dawley rats (6-week-old, male, 160–180 g) were obtained from Vital River Laboratories (Beijing, China). All animals were kept in environments with constant humidity and temperature. The Nanjing University Institutional Animal Care and Use Committee authorized all procedures for using animals in research (IACUC-2003040).

SD rats were fed for 7 days to adapt to the environment and were randomly divided into four groups: control group, sham group, ligature group and ligature + MitoQ@PssL NPs group. Pentobarbital sodium was used to anesthetize the rats, ligatures were firmly inserted sub-gingivally, around the bilateral maxillary second M, and LPS (total 60 μl of 2 mg/ml) was twice weekly injected under the second maxillary molars in the palatal gingivae. MitoQ@PssL NPs (1 mg/ml) were then injected *in situ* with a single dosage of 20 μl per site at each expected time point (total 20 μl of 1 mg/ml, once a day). After 4 weeks, the animals were sacrificed for their maxilla and major organs.

### 2.9 Micro-CT and bone parameter analysis

For the micro-CT examination, all maxilla samples that were taken were dried (viva CT 80, Switzerland). Slice thickness was fixed at 15.6 m, and the X-ray source was set to 70 kV and 113 A. Three-dimensional (3D) images were reconstructed using CTvox software and bone mineral density (BMD) was analyzed using CTAn software. The distance between the alveolar bone crest (ABC) and cementoenamel junction (CEJ) was measured at six sites to indicate the attachment loss (palatal and buccal aspects of the second molars).

### 2.10 Histological evaluation

All maxillary specimens were decalcified in 10% ethylene diamine tetra-acetic acid and preserved in 10% paraformaldehyde solution for pathological investigation (EDTA), dehydrated in gradient alcohol, and embedded in paraffin. The effects of bone formation and bone remodeling were analyzed by H&E staining. Additionally, H&E staining of various organs, such as the heart, liver, spleen, lung, and kidney, was carried out to assess the NPs’ potential *in vivo* toxicity.

### 2.11 Immunohistochemistry

To find out whether Pink1 and Parkin (BOSTER, China) were expressed as proteins in the samples, immunohistochemical staining was used. The slices were incubated with primary antibodies for PINK1 and Parkin (1:100) (BOSTER, China, 1:200) at 4°C overnight after deparaffinization of paraffin-embedded sections. The slices were progressively treated with the second antibody and biotin-labeled horseradish peroxidase (ZSGb Bio, China) after thorough PBS washings for 15 min. Subsequently, the sections were counterstained with hematoxylin (Solarbio, China) after processing with a 3,-3′-diaminobenzidine tetrahydrochloride (DAB) kit (BOSTER, China) in line with the procedure. Inverted microscope (Olympus, Japan) images were taken using neutral resin sealed-slices that were prepared for observation.

### 2.12 *In Vivo* fluorescence imaging of ROS scavenging in the oxidative stress model

BALB/c nude mice were purchased from Vital River Laboratories (Beijing, China), weighting about 20 g were chosen for the establishment of an oxidative stress model, LPS (0.02 ml, 1 mg/ml) daily subgingival injection administration. A total of 3 days later, MitoQ@PssL NPs were injected *in situ* with a dosage of 200 μl per site. A total of 24 h later, the MitoSOXTM Red probe (Yeasen, China) was injected to detect the remaining mtROS. The results of whole-body fluorescence imaging were used an IVIS^®^ Lumina Ⅲ imaging system (PerkinElmer, United States). ImageJ software was used to measure and quantify the fluorescence signal intensity.

### 2.13 Statistical analysis

The data were presented as means ± standard deviations. Statistical analyses were performed with GraphPad Prism 8. One-way analyses of variance (ANOVA) were performed to detect the significant effects of the variables. *p < .05 was considered statistically significant.

## 3 Results

### 3.1 Synthesis and characterization of MitoQ@PssL NPs

**FIGURE 1:**
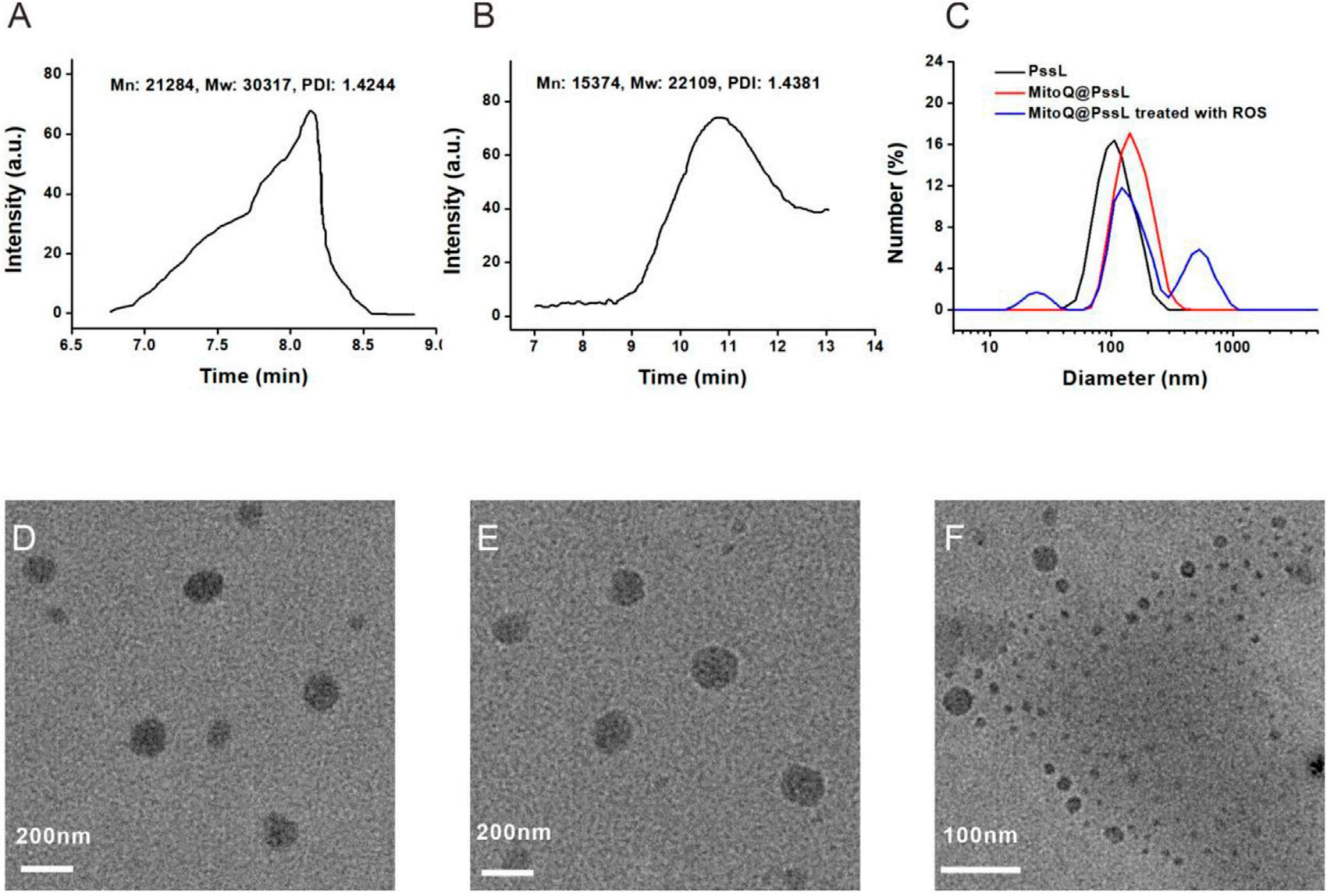
Characterization of MitoQ@PssL NPs. **(A)** GPC of PssL.**(B)** GPC of PssL treated with ROS. **(C)** DLS profiles. **(D)** TEM image of PssL NPs. **(E)** TEM image of MitoQ@PssL NPs. **(F)** TEM image of MitoQ@PssL NPs treated with ROS (0.5 mmol/L H2O2, 4 h).

As described in our previous report, the ROS-responsive amphiphilic polymer PssL was synthesized through the insertion of an ROS-cleavable thioketal bond between hydrophilic polyethylene glycol (PEG) and hydrophobic polycaprolactone (PCL) (Qiu et al., 2021). The molecular weight (Mn: 21284, Mw: 30317) was calculated based on its retention time on the gel column (Figure 1A). However, once treated with ROS, the retention time was remarkably prolonged, along with a substantial decrease in the molecular weight (Mn: 15374, Mw: 22109), revealing the cleavage of amphiphilic PssL in response to ROS stimulation (Figure 1B). As an amphiphilic polymer, PssL may be used as a robust drug delivery carrier for periodontitis drugs (such as MitoQ) through 40-min sonication-assisted self-assembly. Transmission electron microscopy (TEM) was employed to investigate the morphology of the self-assembled NPs. As shown in Figures 1D,E, the encapsulation of MitoQ into PssL NPs (MitoQ@PssL NPs) slightly increased the mean diameter from ∼85 nm to ∼115 nm; however, no change in the homogeneously dispersed spherical morphology was observed. Notably, once exposed to ROS, the morphology of MitoQ@PssL NPs was destroyed completely (Figure 1F), resulting in two extra impurity peaks in the dynamic light scattering (DLS) results (Figure 1C) that indicated the breakage of PssL and the release of MitoQ.

Prior to the usage of MitoQ@PssL NPs as ROS scavengers in periodontal disease both *in vitro* and *in vivo*, we explored their cytotoxicity and blood compatibility. The CCK-8 assay was performed to determine the relative viability of hPDLSCs after exposure to MitoQ@PssL NPs (Supplementary Figure S2). As expected, no significant cytotoxicity was induced by NPs in any of the groups, even at a high concentration of 1 μg/ml. The haemolytic assay was conducted to determine blood compatibility, as shown in Supplementary Figure S3. A statistically significant difference in the haemolysis rate was not observed after a coincubation of MitoQ@PssL NPs and blood components for 3 h, even at a high concentration of 200 ng/mL. As shown in Supplementary Figures S4, S5, MitoQ@PssL NPs exhibited good biocompatibility and the uptake of NPs did not damage the cell membrane or mitochondrial function. The apoptosis assay (Supplementary Figure S6) indicated that NPs did not induce significant apoptosis. Calcein-AM/propidium iodide (PI) probes were used for double staining of live/dead cells, and the results showed that NPs with high biocompatibility did not alter the morphology and relative viability of hPDLSCs (Supplementary Figure S7), as red-stained cells were rarely observed. As mentioned above, all of these necessary experiments indicated the overall safety of MitoQ@PssL NPs *in vitro*, even at concentrations up to 1,000 ng/ml.

### 3.2 MitoQ@PssL NPs induced mitophagy through the PINK1-Parkin pathway in an LPS-induced inflammatory injury model

The good biocompatibility of MitoQ@PssL NPs was observed. Next, the essential role of NPs in protecting hPDLSCs against LPS-induced inflammatory injury by inducing mitophagy was further verified. As illustrated in Supplementary Figure S8, LPS led to a significant decrease in cell viability and an increase in LDH release, and MitoQ@PssL NPs markedly mitigated the changes in these indicators. The mitophagy inhibitor cyclosporin A (CSA) reduced cell viability and increased LDH release from LPS-treated hPDLSCs, while the mitophagy agonist carbonyl cyanide 3-chlorophenylhydrazone (CCCP) exerted the opposite effects, suggesting that stimulating mitophagy visibly protected against LPS-induced inflammatory injury. Moreover, MitoQ@PssL NPs increased the dissipation of the mitochondrial membrane potential (ΔΨm) induced by LPS. Sequential treatment with CSA and MitoQ@PssL NPs attenuated the effect of LPS on enhancing ΔΨm, suggesting that MitoQ@PssL NPs modulated mitochondrial function *via* mitophagy (Supplementary Figure S9).

**FIGURE 2:**
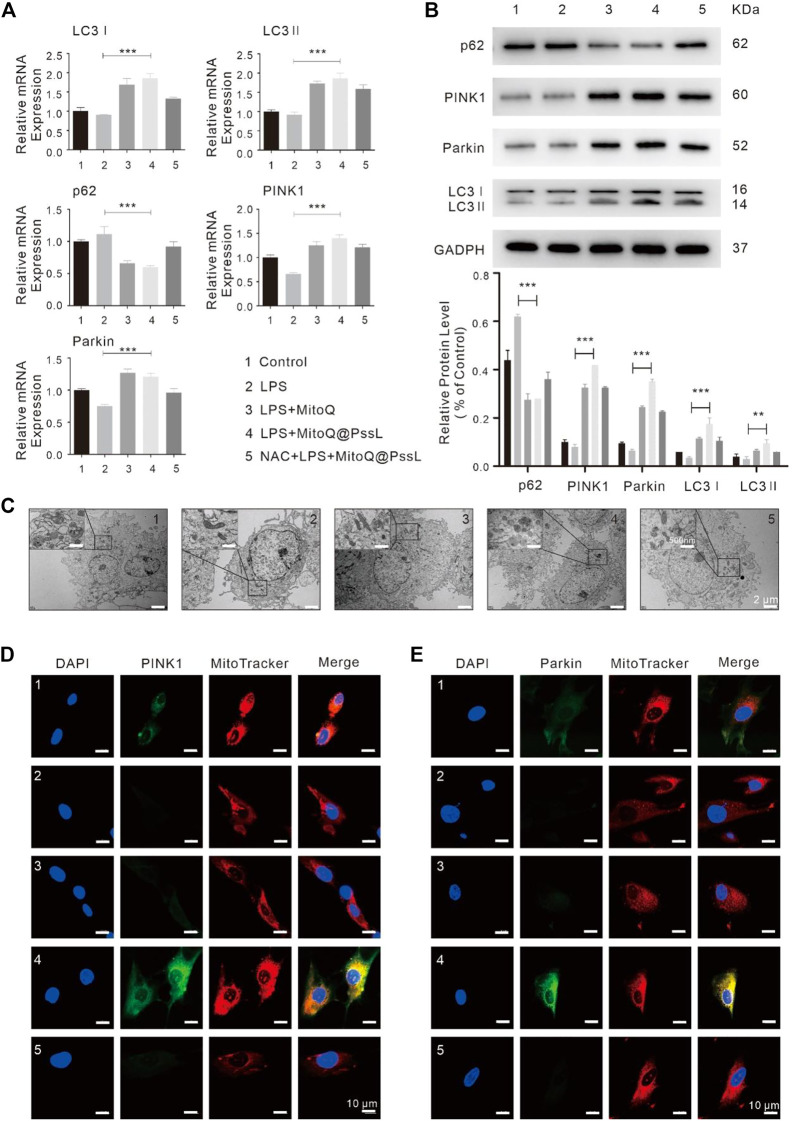
MitoQ@PssL NPs significantly promoted mitophagy and upregulated the PINK1-Parkin pathway in LPS-induced hPDLSCs (1: Control, 2: LPS, 3: LPS + MitoQ, 4: LPS + MitoQ@PssL NPs, and 5: NAC + LPS + MitoQ@PssL NPs). **(A)** The expression of PINK1, Parkin, LC3I, LC3 II and p62 were detected using RT-PCR. **(B)** The expression of PINK1, Parkin, LC3I, LC3II and p62 were detected using western blot analysis. **(C)** Representative TEM images of mitochondrial morphology in hPDLSCs. Scale bar: 2 μm. Partial enlargements were shown in the black boxes. Scale bar: 500 nm. Mitochondrial morphology and the colocalization of the mitochondrial with **(D)** PINK1 **(E)** Parkin were analyzed using a 63× oil immersion lens (630×). Scale bar = 10 nm. (* means *p* < .05, ** means *p* < .01, *** means *p* < .001).

In addition, we investigated mitophagy-associated mRNA and protein expression by performing RT‒PCR and western blot analyses. The RT‒PCR analysis showed that LPS led to a marginal decrease in the expression of specific mitophagy markers, including LC3, PINK1, and Parkin, compared with normal hPDLSCs. In contrast, MitoQ@PssL NPs substantially increased the expression of these mitophagy markers in LPS-pretreated hPDLSCs (Figure 2A). Western blot analyses also confirmed that LC3Ⅰ, LC3Ⅱ, PINK1 and Parkin protein levels were significantly increased upon MitoQ@PssL NP treatment, while p62 protein levels were visibly decreased (Figure 2B). The LC3II/LC3I ratio is assumed to be an index of autophagosome and autolysosome accumulation in cells. The increased conversion of LC3I to LC3II is considered a marker for the initiation of autophagy because of its aggregation and localization in autophagosomes. Notably, the production of LC3Ⅱ in LPS-treated hPDLSCs was facilitated by MitoQ@PssL NPs, indicating autophagy activation. Furthermore, a large number of autophagosomes was observed in hPDLSCs treated with 200 ng/ml MitoQ@PssL NPs using TEM (Figure 2C).

The trends for the expression of LC3 and p62 (markers of autophagosomes), as well as PINK1 and Parkin (markers of the PINK1-Parkin pathway of mitophagy) indicated that MitoQ@PssL NP treatment further induced mitophagy in LPS-treated hPDLSCs. However, following treatment with a mix of N-acetylcysteine (NAC, a classical antioxidant) and MitoQ@PssL NPs, the colocalization of the mitochondria with PINK1 and Parkin was remarkably constrained (Figures 2D, E). Considering that NAC was a classic antioxidant, we used NAC as the gold standard for the complete elimination of ROS. Theoretically, the complete elimination of ROS may be conducive to the construction of anti-inflammatory microenvironment and tissue regeneration, but the result was not the same. Compared with the MitoQ@PssL NPs group (Partially ROS clearing), the state that ROS is not completely eliminated seems to be more conducive to the formation of the tissue regeneration environment. Mitophagy was also detected by performing immunofluorescence staining. Colocalization of PINK1 and Parkin with MitoTracker indicated the reduced formation of mitophagosomes after pretreatment with NAC. TEM images of mitochondrial morphology and autophagosomes also suggested that NAC reversed the effect of MitoQ@PssL NPs on the damaged mitochondrial morphology caused by LPS injury (Figure 2C). These results confirmed that MitoQ@PssL NPs protected against LPS-induced inflammatory injury by increasing mitophagy, and this process may be related to the regulation of mtROS production.

### 3.3 Effect of MitoQ@PssL NPs on the behaviour of inflammatory cells

Based on our understanding of mitophagy induced by MitoQ@PssL NPs, we then evaluated their ability to remove ROS. We exposed hPDLSCs to LPS to simulate an inflammatory environment and then assessed the effect of MitoQ@PssL NPs on regulating intracellular and mitochondrial ROS levels.

The expression of TNF-α and IL-1β in LPS-treated hPDLSCs is shown in Supplementary Figure S10. The biomarkers of the level of periodontal inflammation revealed that MitoQ@PssL NPs efficiently assuaged the activation of these inflammatory mediators and efficiently attenuated both intracellular and mitochondrial ROS levels in hPDLSCs after LPS treatment (Supplementary Figure S11). The intracellular ROS level was determined by performing staining using DCFH-DA as a fluorescence probe, and MitoSOX™ Red reagent was used along with fluorescence microscopy to visualize the process of superoxide production in mitochondria, which can also be used for the quantitative detection of mitochondrial ROS levels. After the cells were incubated with LPS to stimulate the generation of intracellular and mitochondrial ROS, hPDLSCs were incubated with or without NPs. The groups were as follows: Control, LPS, LPS + MitoQ, LPS + MitoQ@PssL NPs, and NAC + LPS + MitoQ@PssL NPs. As shown in Supplementary Figure S11, the fluorescence intensity of intracellular ROS was consistent with that of mtROS, and these results were consistent with the theory that ROS in histocytes are a byproduct of the mitochondrial respiratory chain, which are regarded as the main source of ROS in histocytes.

**FIGURE 3:**
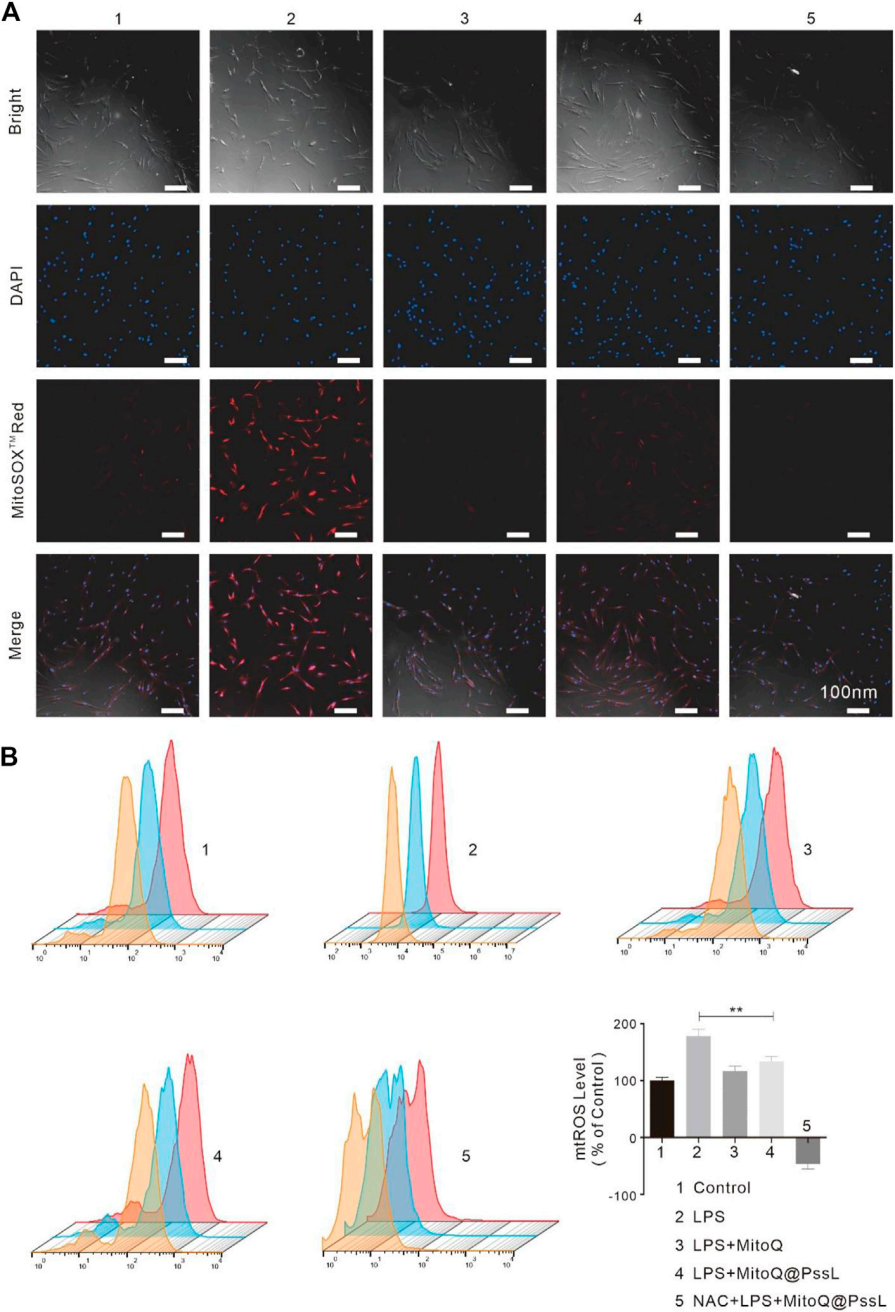
ROS regulation of hPDLSCS treated with MitoQ@PssL NPs (1: Control, 2: LPS, 3: LPS + MitoQ, 4: LPS + MitoQ@PssL NPs, and 5: NAC + LPS + MitoQ@PssL NPs). **(A)** Typical fluorescence images of hPDLSCs. **(B)** Flow cytometry and quantitative measurement of mtROS formation (MitoSOXTMRED) of hPDLSCs. (** means *p* < .01).

Combined with the results from the aforementioned studies, the process by which MitoQ@PssL NPs modify mitophagy may be intimately associated with the adjustment of mtROS production. LSCM observations together with flow cytometry were used to detect the mtROS removal capacity of MitoQ@PssL NPs. As shown in Figure 3A, unlike the antioxidant (MitoQ and NAC)-treated group, the MitoQ@PssL NPs slightly decreased the fluorescence intensity in the LPS-treated group. As shown in Figure 3B, the flow cytometry results also suggested that approximately 62.5% of mtROS were removed by MitoQ@PssL NPs upon coincubation at a concentration of 200 ng/ml. Based on these results, MitoQ@PssL NPs act as potent ROS scavengers to protect hPDLSCs from damage by reducing excess mitochondrial oxidative stress induced by LPS in the inflammatory environment.

Previous studies have confirmed that mitophagy is jointly associated with the maintenance of mitochondrial function, and mitochondrial dysfunction is associated with mitochondrial oxidative stress caused by excess mtROS levels (Nhu et al., 2021). In addition to antioxidants (NAC and MitoQ), MitoQ@PssL NPs promote mitophagy because our designed drugs intelligently respond to mtROS and slow the release of MitoQ, which maintains mtROS at a low level rather than completely clearing them.

### 3.4 Effects of MitoQ@PssL NPs on the osteogenic differentiation of LPS-induced hPDLSCs

**FIGURE 4:**
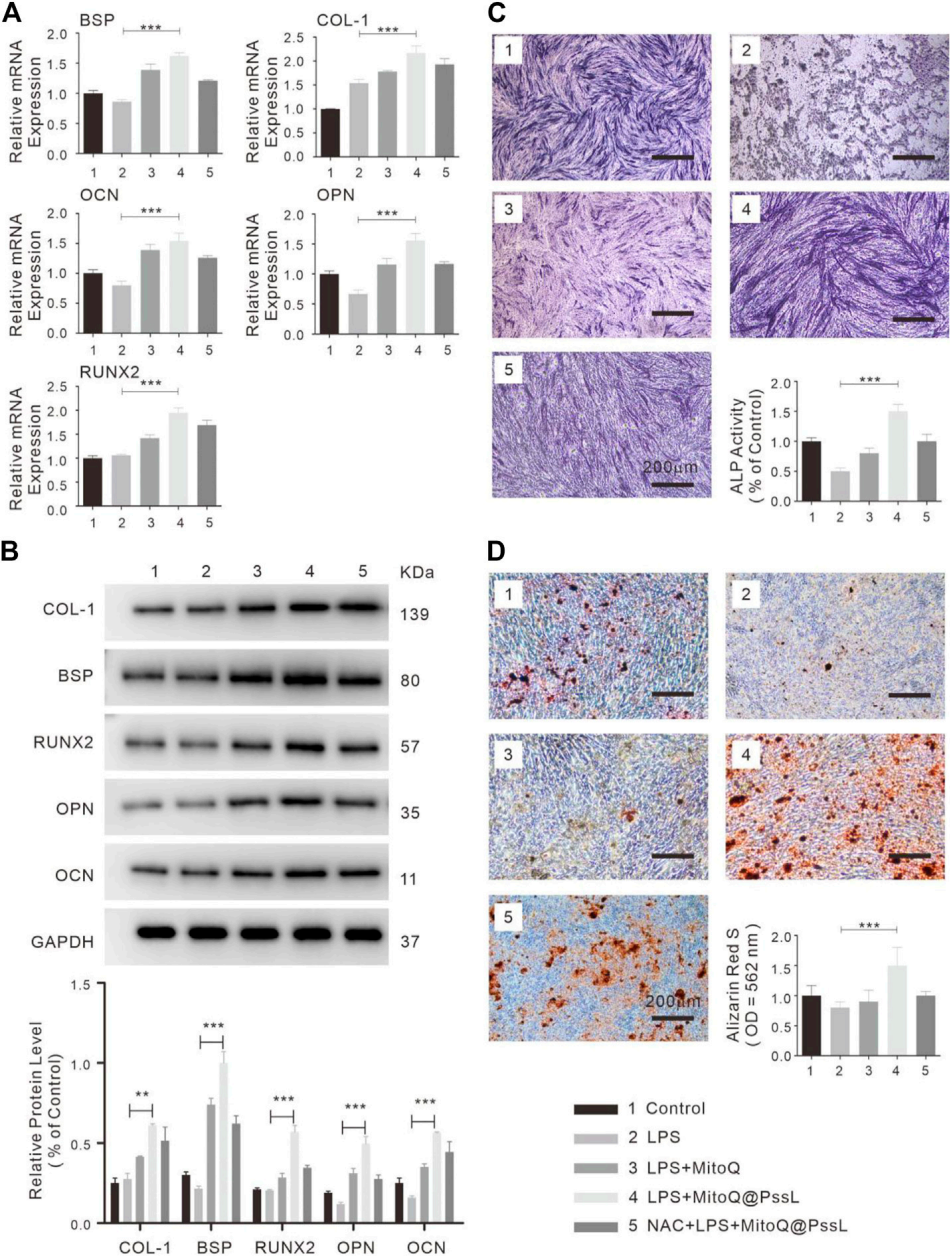
The osteogenic differentiation of hPDLSCs treated with MitoQ@PssL NPs (1: Control, 2: LPS, 3: LPS + MitoQ, 4: LPS + MitoQ@PssL NPs, and 5: NAC + LPS + MitoQ@PssL NPs). **(A)** Osteogenic related gene expression on day 7. **(B)** Osteogenic related protein experssions of hPDLSCs on day 14 after exposed to LPS with pretreatment of Control, LPS, LPS + MitoQ, LPS + MitoQ@PssL NPs, and NAC + LPS + MitoQ@PssL NPs. **(C)** ALP staining and related activity of hPDLSCs on day 7. **(D)** ARS on day 14. (** means *p* < .01, *** means *p* < .001).

The regenerative capability of hPDLSCs may be significantly reduced in the periodontitis microenvironment. The detection of the function of MitoQ@PssL NPs in the osteogenic differentiation of hPDLSCs was further evaluated by performing RT‒PCR, analyses of relative ALP activity and staining, alizarin red staining (ARS), and western blot analyses. As shown in Figure 4A, the expression levels of osteogenesis-related genes (BSP, OCN, and OPN) indicated that the capacity of hPDLSCs for osteogenic differentiation was significantly suppressed upon exposure to LPS; however, MitoQ@PssL NPs definitely enhanced the differentiation ability to a greater extent even than the control group. The protein expression levels of osteogenic markers (BSP, COL-1, OCN, OPN, and Runx2) were also increased in the presence of MitoQ@PssL NPs (Figure 4B). Moreover, the analysis of both ALP staining and ALP activity levels (Figure 4C) on Day 7 showed that LPS decreased the osteogenic differentiation capacity of hPDLSCs, while MitoQ@PssL NPs reversed the suppression. The cells in culture dishes were stained with ARS to detect the calcium deposits in the extracellular space and monitor the mineralization associated with the osteogenic differentiation of hPDLSCs. Mineralization was clearly detectable after a minimum culture time of 14 days in differentiation medium. Representative images and relative quantification in Figure 4D show that the maximal osteogenic differentiation was promoted by 200 ng/ml MitoQ@PssL NPs. These results presented a consistent trend of the ability of MitoQ@PssL NPs to enhance the osteogenic differentiation of the LPS-induced inflammatory model in our study. In summary, MitoQ@PssL NPs at a concentration of 200 ng/ml promoted the osteogenesis of LPS-induced hPDLSCs.

### 3.5 MitoQ@PssL NPs inhibited oxidative stress

**FIGURE 5:**
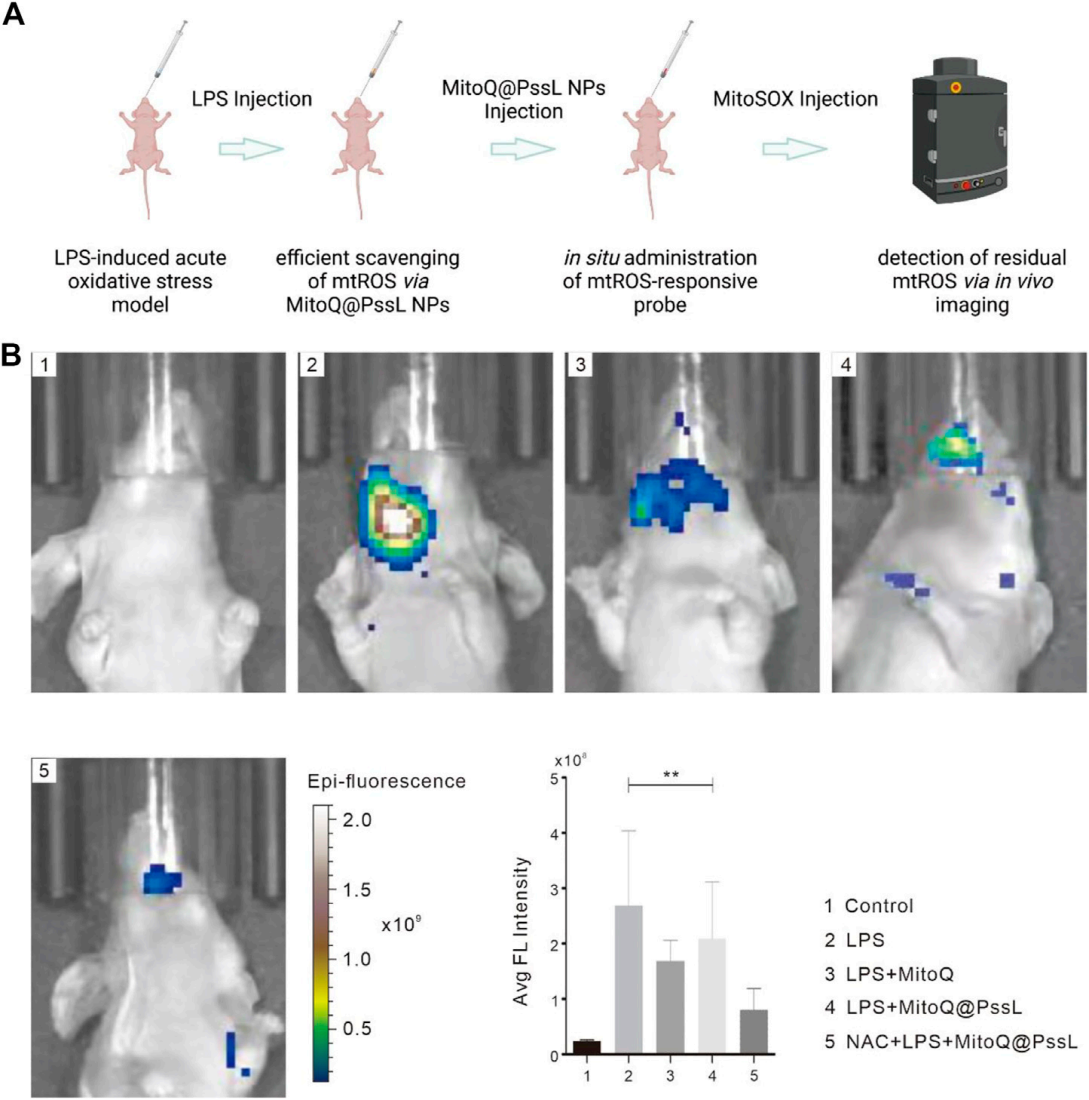
Scavenging efficiencies of LPS-induced acute oxidative stress model in BALB/c nude mice. **(A)** Schematic illustration of LPS-induced periodontal disease in BALB/c nude mice and relative experimental design of mtROS scavenging. **(B)** Fluorescence imaging of mtROS scavenging capacity and relative quantitative information. (** means *p* < .01).

The acute mouse oxidative stress model was established by administering a local injection of LPS into gingivae, and the effect of MitoQ@PssL NPs on eliminating inflammation and local mtROS production *in vivo* was detected using an *in vivo* imaging instrument to again confirm our finding of efficient mtROS removal by MitoQ@PssL NPs that served as scavengers (Figure 5). As expected, a high fluorescence signal was observed in the LPS-treated group, while no fluorescence signals were detected in the control group. According to our observations, hPDLSCs pretreated with both MitoQ and NAC showed a relatively lower fluorescence signal than those from the LPS-treated group, indicating that representative antioxidants (MitoQ and NAC) efficiently removed mtROS. Noticeably, the difference in fluorescence intensity of the MitoQ@PssL NPs-treated group compared with the LPS and antioxidant-exposed groups implied that mtROS levels were reduced to some extent by MitoQ@PssL NPs but were not completely cleared. Additional quantitative analyses using ImageJ software confirmed our results.

### 3.6 MitoQ@PssL NPs accelerated alveolar bone osteogenesis in the periodontitis rat model

The therapeutic effect of MitoQ@PssL NPs on the rat periodontitis model was studied by assessing alveolar bone loss and pathological changes in periodontal tissue. The experimental periodontitis model of rats was established by wire ligation. All rats in the ligature-induced group were anesthetized with an inhalation method using isoflurane for periodontal disease induction. The ligatures were placed using a sterilized silk thread tied around the cervix of the second maxillary molars, knotted at the palatal surface of the second M (Supplementary Figure S12), and then LPS (total 60 μl of 2 mg/ml) was injected twice a week into the palatal gingivae under second maxillary molars. Prior to the development of an animal model, an explore of long-term toxicity, a series of *in vivo* experiments were designed and carried out. Observation of body weight was the most-straight forward method of evaluating the toxicity of MitoQ@PssL NPs (Supplementary Figure S13). Excessive pro-inflammatory cytokines and some inflammatory mediators were found in periodontal disease including TNF-α, IL-6, and IFN-γ could quantify the acute or chronic inflammatory responses after the administrations of MitoQ@PssL NPs (Supplementary Figure S14). Histological analysis of exposed tissues could determine the tissue damages from toxic exposure induced by the nanoparticles (Supplementary Figure S15).

**FIGURE 6:**
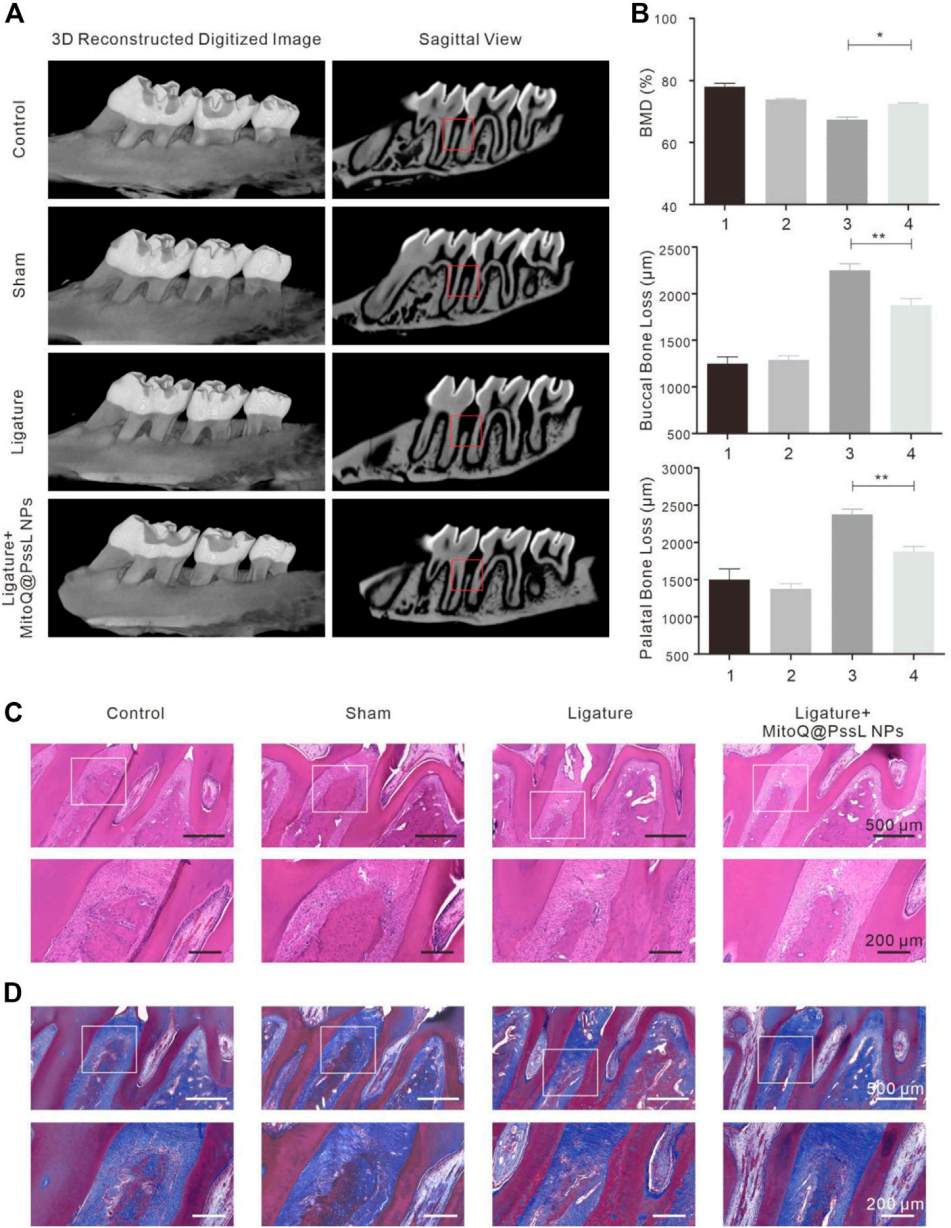
MitoQ@PssL NPs accelerated alveolar bone osteogenesis in periodontitis rat model. **(A)** Micro-CT reconstruction images of samples and bucco-palatal section images of the maxillary molars treated with different experimental conditions. **(B)** Quantitative measurements of alveolar bone loss of both buccal and palatal areas. **(C)** Histological analysis by H&E and **(D)** Masson’s trichrome staining of the interdental area between the upper first and second M. Scale bar: 500 μm. A partial enlargement is shown in the black box. Scale bar: 200 μm. (* means *p* < .05, ** means *p* < .01).

Alveolar bone loss was imaged using micro-CT (Figure 6A), and alveolar bone loss data were quantified by measuring the distance of CEJ-ABC at each point of the maxillary second molar (Figure 6B). The levels of bone loss and bone mineral density (BMD, measure the density range in the red box) indicated that bone resorption was inhibited by injecting MitoQ@PssL NPs. In the 3D reconstructed alveolar bone diagram, the height of alveolar bone was lost, and M2 furcation was exposed in the ligature group. The statistical analysis of the distance of CEJ-ABC specified that ligation decreased the height of alveolar bone at six sites compared with the control group, while MitoQ@PssL NP treatment reduced the extent of bone loss at six sites.

H&E staining and Masson’s trichrome staining were performed to further verify the pathological changes in periodontal tissue (Figures 6C,D). The attachment height of periodontal fibers and the shape of the bone structure in the control group were normal. The sham operation group also displayed no damage to periodontal fibers and alveolar bone, while fibers were degenerated and degraded along with inflammatory cell infiltration in the ligature group. After continuous treatment with MitoQ@PssL NPs for 4 weeks, the loss of alveolar bone height was attenuated compared with the ligature group, indicating that MitoQ@PssL NPs inhibited the inflammatory resorption of alveolar bone in a periodontitis model.

### 3.7 MitoQ@PssL NPs significantly promoted mitophagy in periodontitis rat model

**FIGURE 7:**
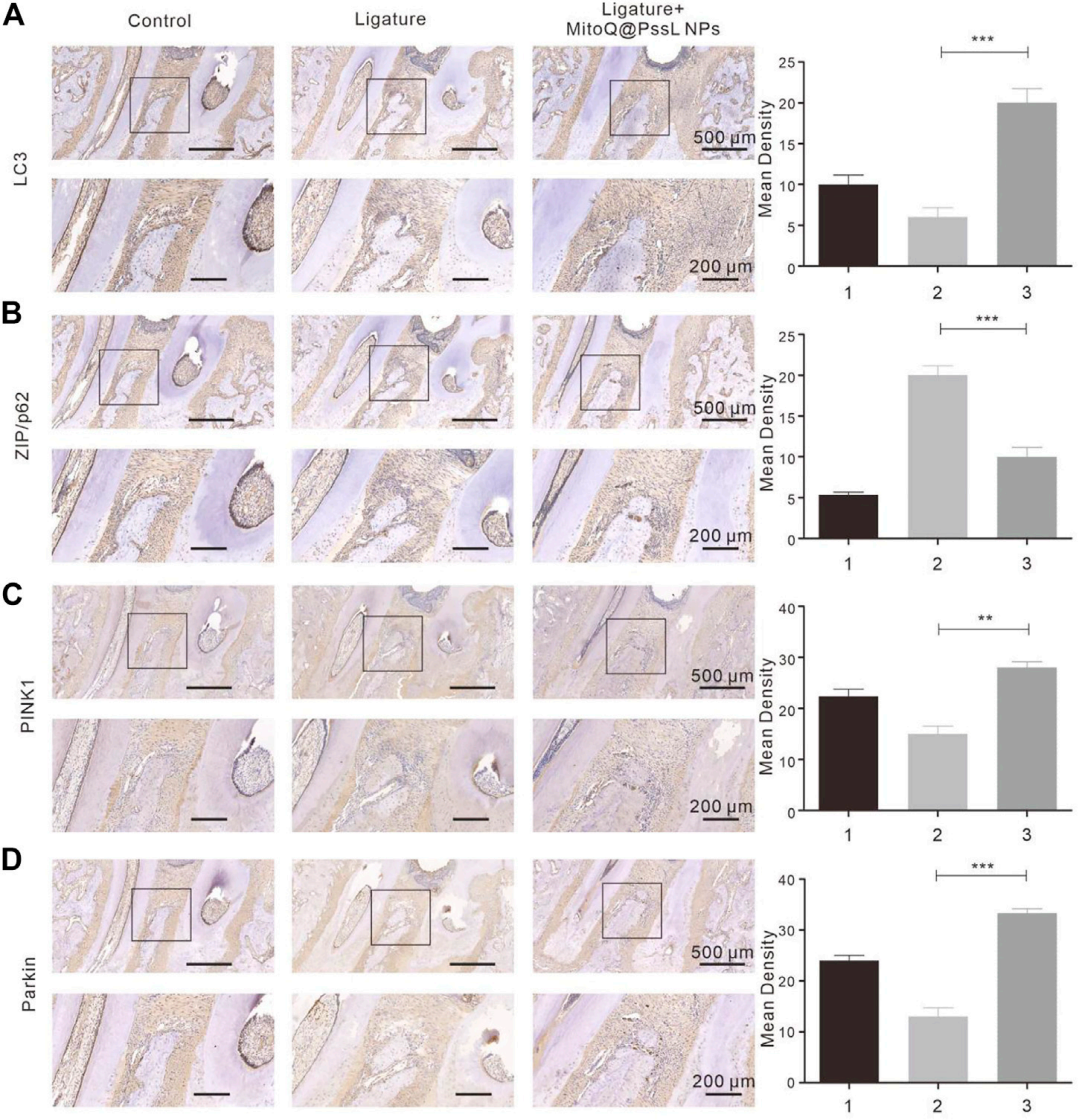
The expressions of **(A)** LC3, **(B)** ZIP/p62, **(C)** PINK1, **(D)** Parkin were detected using immunohistochemistry (50×, 100×). Partial enlargements were shown in the black boxes. 50× Scale bar: 500 μm. 100× Scale bar: 200 μm. The corresponding quantitative analysis of LC3, ZIP/p62, PINK1, Parkin expression in immunohistochemical staining images. (** means *p* < .01, ** means *p* < .001).

Precultured with LPS resulting in a strong inhibition of mitophagy as shown by the immunohistochemical analysis (Figure 7). This was further confirmed by the lack of interaction between PINK1 and Parkin in the NAC-supplemented group. Immunohistochemical analysis indicated that MitoQ@PssL NPs significantly promoted the expression of PINK1 (Figure 7C) and Parkin (Figure 7D). Compared with the periodontitis rats, the antibody expression (PINK1 and Parkin) was significantly up-regulated in the MitoQ@PssL NPs treated rats. In addition, the antibody expression in the control and sham group were remain relatively lower, which was also in line with our expectations.

## 4 Discussion

Periodontitis is a highly prevalent global health problem that negatively affects the quality of life of many populations (Sui et al., 2020). Antioxidant therapy has been considered an effective strategy to treat periodontal disease caused by oxidative stress (Zhang et al., 2018). Therefore, ROS scavengers are excellent choices for the treatment of periodontal diseases (Kang et al., 2020). In recent years, nanotechnology has provided new insights into the development of more effective ROS scavengers, which have emerged as a promising strategy for the treatment of periodontitis (Román-Malo and Bullon, 2017). Nanomaterial-based antioxidants effectively scavenge ROS, prevent ROS-mediated tissue damage, and relieve inflammation in individuals with periodontitis. Studies have confirmed that excess ROS production in periodontitis leads to systemic arterial endothelial dysfunction, which is a potential risk factor for atherosclerosis (Fuentes et al., 2019). The administration of exogenous antioxidants scavenges excess ROS from periodontal tissues and reduces inflammation. Several research reports (Sun et al., 2017; Wang et al., 2020; Marek-Iannucci et al., 2021) have suggested that excess cellular ROS and analogous oxidative stress injury usually activate acute autophagy-associated pathways, which consecutively alleviate oxidative stress by reducing ROS levels. Whether autophagy-enhancing could stably reduce ROS production and the consequent mitochondrial damage, there were still few studies to confirm, and the mechanism of autophagy in tissue regeneration has been supported. To some extent, researchers have tried to synthesize bionic autophagy enhancers that activate autophagy (Giordano et al., 2013; Zhang et al., 2019), reduce the cellular ROS levels and certainly reduce oxidative stress-induced damage and preserve cell functions, including cell proliferation, cell metabolism and differentiation (Sharma et al., 2018; Piscianz et al., 2021; Yang et al., 2021). However, few reports have described the use of effective autophagy enhancers to treat periodontitis that stabilize the level of autophagy activation and facilitate cell survival. In periodontal therapy, antioxidant, anti-inflammatory and regenerative treatments are all integral. Hence, the development of feasible autophagy-enhancing materials is very important to treat periodontitis. Autophagy-enhancing materials used in the periodontitis microenvironment not only must have the characteristics of basic drug delivery nanomaterials, such as precise delivery, low toxicity, high biocompatibility and stable physicochemical properties (Yamada et al., 2020), but also must promote the regeneration of periodontal tissue by activating autophagy while stabilizing the local redox balance.

After considering these properties, we first explored MitoQ@PssL NPs as a safe therapeutic for periodontitis by developing ROS-responsive nanoparticles that release MitoQ. In this nanosystem, an autophagy enhancer named MitoQ, is encapsulated by the ROS-responsive amphiphilic polymer polyethylene glycol-thioketal bond-polycaprolactone (PssL). As depicted in Figure 1A, PssL was synthesized as described in our previous report, and the encapsulation of MitoQ was achieved by an oil/water emulsification method. Under serious oxidative stress conditions, extremely high ROS levels were detected, resulting in the cleavage of PssL and subsequent release of MitoQ (Figures 1C,F), which effectively cleared excess ROS after a local injection into periodontal tissue. This reaction is terminated after the ROS concentration decreases to an appropriate level. The remaining unreacted MitoQ@PssL NPs may also be stably stored, ensuring that they also play an effective role in ameliorating the next increase in ROS levels.

We found that MitoQ@PssL NPs prevented increased oxidative stress and activated mitophagy, both of which contribute to osteogenesis and remodeling in periodontitis. Mitophagy has been shown to be a protective process in the pathogenesis of inflammatory diseases. (Jin et al., 2019). Enhanced mitophagy is also linked to the consumption of excessive ROS and the subsequent protection of cells. Interestingly, MitoQ@PssL NPs exerted anti-inflammatory and cytoprotective effects by activating mitophagy, as evidenced by the increased production of LC3, PINK1 and Parkin, the decrease in p62 levels, and the induction of mitophagy-related vesicle formation in hPDLSCs. These findings were verified by gene and protein expression, as well as the observation of cell structures under TEM (Figure 2). Treatment with the mitophagy inhibitor NAC reversed the anti-inflammatory effect of MitoQ@PssL NPs, as evidenced by increased production of IL-1β and TNF-α (Supplementary Figure S10). Based on these results, the ROS-scavenging effects of MitoQ@PssL NPs might be related to mitophagy activation. We found that mitophagy effectively alleviated oxidative stress and reduced both intracellular and mitochondrial ROS levels, as observed in CLSM images (Supplementary Figure S11; Figure 3), which was beneficial for protecting hPDLSCs from oxidative stress (Figure 5), inflammatory reactions, and bone degeneration. These results were further verified in the rat periodontitis model (Figure 6 and Figure 7). Fluorescence imaging of the ROS removal capacity and relative quantitation indicated that MitoQ@PssL effectively reduced the oxidative stress caused by LPS. MitoQ@PssL NPs inhibited chronic inflammatory bone destruction due to ligature and LPS in rat periodontitis models. Long-term toxicity was investigated by monitoring changes in body weight (Supplementary Figure S13), performing a histological analysis of the main organs (Supplementary Figure S14), and analyzing the levels of inflammatory factors (Supplementary Figure S15), indicating that 2 mg/ml MitoQ@PssL NPs was a safe and efficient dose for local subgingival injection.

Although accumulating evidence has indicted a link between mitophagy and the differentiation capacity of stem cells in human health and disease (Yao et al., 2019b; Kim et al., 2021b), few studies have reported the key components through which mitophagy regulates the differentiation capacity of hPDLSCs in periodontitis and *vice versa*. Here, we designed and constructed efficient nanoparticles encapsulating a mitophagy enhancer for mtROS removal in oxidative stress-induced periodontal disease using MitoQ@PssL NPs as intelligent periodontitis microenvironment-controlled drug release nanoparticles. These nanoparticles promoted the osteogenic differentiation of hPDLSCs by activating the PINK1-Parkin-related mitophagy pathway. A series of systemic investigations were performed in this study to improve our comprehensive understanding of the mitophagy-inducing and antioxidant effects of MitoQ@PssL NPs and their related mechanisms. The balanced redox-mitophagy pathway activated by MitoQ@PssL NPs may contribute to protecting host immunity and preventing the process of tissue destruction in individuals with periodontitis, thus maintaining periodontal bone tissue reconstruction. We expect that this study represents a breakthrough in the treatment of periodontitis. As for the regulatory mechanism of mtROS and bone metabolism. The current results only confirm that mtROS is involved in the regulation of bone formation through gene and protein levels, and may be related to PINK1-Pakin-related mitophagy pathway. There is still a lack of experimental data support for mtROS and bone destruction. Although there are many studies in this part and there has been a large amount of literature support, but considering the complexity of bone metabolism, We hope to further study the mechanism of mtROS and bone metabolism and enrich scientific data based on this study in the future. Considering the current experiment is still superficial in the mechanism research and lacks more in-depth research results, we hope that the molecular mechanism of mtROS and periodontal bone metabolism can be further discussed based on this research in the future.

## 5 Conclusion

In conclusion, as a novel mitophagy enhancer, MitoQ@PssL NPs exert excellent anti-inflammatory effects by activating the PINK/Parkin signaling pathway to induce mitophagy and partially adjust mitochondrial ROS levels, thereby accelerating osteogenesis in subjects with periodontitis. These findings imply that mitophagy-induced MitoQ@PssL NPs with an excellent negative feedback regulatory system might be a potential therapeutic option in periodontal tissue engineering and the treatment of periodontitis while providing additional insights into inflammatory diseases.

## Data Availability

The original contributions presented in the study are included in the article/Supplementary Material, further inquiries can be directed to the corresponding authors.
