# Supplementary material for: Robust intervention for oxidative stress-induced injury in periodontitis via controllably released nanoparticles that regulate the ROS-PINK1-Parkin pathway
Source: Front Bioeng Biotechnol. 2022 Dec 15;10:1081977. doi: 10.3389/fbioe.2022.1081977 (PMC9798290; doi:10.3389/fbioe.2022.1081977)
Supplement: Supplementary file 1 [file DataSheet1.pdf]

Supporting Information

A robust intervention for oxidative stress-induced injury in periodontitis *via* controllably released nanoparticles that regulate the ROS-PINK1-Parkin pathway

Xincong Li[1], Yue Zhao[1], Haoran Peng[1], Deao Gu[2], Chao Liu[2*], Shuangshuang Ren[1*], and Leiying Miao[1*]

[*]Correspondence:

dxliuchao@163.com; zjndrenss@163.com; miaoleiying80@163.com

[1]Department of Cariology and Endodontics, Nanjing Stomatological Hospital, Medical School of Nanjing University, Nanjing, Jiangsu 210008, China.

[2]Department of Orthodontics, Nanjing Stomatological Hospital, Medical School of Nanjing University, Nanjing, Jiangsu 210008, China.

Cytotoxicity analysis of MitoQ@PssL NPs

hPDLSCs were seeded in 96-well plates for 24 h, and the culture medium was then replaced with a fresh medium containing different concentrations of MitoQ@PssL NPs. A cell counting kit-8 (CCK-8, Keygen, China) was used to evaluate cell viability at 24 h and 48 h and the cell viability percentages were calculated. After the cells were incubated with MitoQ@PssL NPs for 48 h, the cells were washed with PBS and live and dead cells were stained with Calcein-AM/Propidium Iodide (CAM/PI) (Solarbio, China). Fluorescence photographs were collected using a confocal fluorescence microscope (LSCM, A1R, Nikon, Japan). LDH release (Biotechnology, China) and ATP levels (Solarbio, China) were detected according to the manufacturer's instructions. For hemolysis determination, red blood cells were isolated and diluted with 0.9% NaCl solution to obtain a 2% erythrocyte solution. Water was used as a positive control and 0.9% NaCl solution containing different concentrations of MitoQ@PssL NPs were used as experimental samples (n = 3). A total of 3 h later, the absorbance of supernatants at 541 nm was monitored on a UV−vis spectrometer (n = 3). Cell apoptosis was evaluated using the FITC Annexin V Apoptosis Detection Kit I (Keygen, China). In brief, cell samples were washed and centrifuged three times, resuspended in a binding buffer, and stained with 1 μL FITC Annexin V and PI for 15 min in the dark for flow cytometry (BD FACSVerse, USA).

Detection of intracellular ROS level induced by LPS

hPDLSCs were seeded onto glass coverslips in 12-well culture plates to ensure approximately 70% confluency. When hPDLSCs were reached near 80%, the LPS was added to the medium at 8 μg/l for 2 h, then 200 ng/ml MitoQ@PssL NPs were added in the medium and cultured for 24 h. The cells were then treated with a DCFH-DA probe (Keygen, China) and observed under a fluorescence microscope (LSCM, A1R, Nikon, Japan). The quantitative analysis of ROS levels was performed using the ImageJ program.

Expression of Inflammatory Mediators in LPS-Treated hPDLSCs.

hPDLSCs were plated in 6-well plates with a density of 10^6 per well for 24 h. After the medium was removed, cells were incubated in DMEM containing FBS (10%) and LPS(8 μg/l). MitoQ@PssL NPs were then added into above medium with a final concentration of 200 ng/mL. A total of 6 h later, levels of TNF-α and IL-1β in supernatants were measured via an enzyme-linked immunosorbent assay (n = 3).

Long-Term Toxicity.

SD rats, after ligatures and the subgingival injection of MitoQ@PssL NPs and LPS (10 μL of 2 mg/mL), were defined as the test group, and rats without any treatment was denoted as the control group, respectively. Rats body weights of above two groups were recorded for 60 days (n = 6). Blood was collected at each expected time point, and serum was then isolated. Levels of TNF-α, IFN-γ, and IL-6 were measured via an enzyme-linked immunosorbent assay (n = 4).

Table S1 The sequences of primers

Gene（human）	Primer sequence
BSP	Forward:GCACCAGTACCAACAGCACAGA
	Reverse:TTCTGCATTGGCTCCAGTGACA
COL-1	Forward:ACTGGTGATGCTGGTCCTGTTG
	Reverse:ACCATCGTGAGCCTTCTCTTGAG
RUNX2	Forward: CCCAGGCAGTTCCCAAGCATTT
	Reverse:GGTAGTGAGTGGTGGCGGACAT
OCN	Forward:GGCAGCGAGGTAGTGAAGAGAC
	Reverse: GGTCAGCCAACTCGTCACAGTC
OPN	Forward: CGCAGACCTGACATCCAGTACC
	Reverse: TCCCACGGCTGTCCCAATCA
LC3 I	Forward: GCCTTCTTCCTGCTGGTGAACC
	Reverse: AAGCCGTCCTCGTCTTTCTCCT
LC3 II	Forward: GAGAAGCAGCTTCCTGTTCTGG
	Reverse: GTGTCCGTTCACCAACAGGAAG
SQSTM1/p62	Forward: CCCTCTCCCAGATGCTGTCCAT
	Reverse: GCCGCTCCGATGTCATAGTTCT
PINK1	Forward: GCCCGAGTAGCCGCAAATGT
	Reverse: GAGGAGCCAGCCAACCATCTTG
Parkin	Forward: GGAGGTGGTTGCTAAGCGACAG
	Reverse：TGCTCTGCTGATCCAGGTCACA
GAPDH	Forward：AGATCATCAGCAATGCCTCCT
	Reverse：TGAGTCCTTCCACGATACCAA

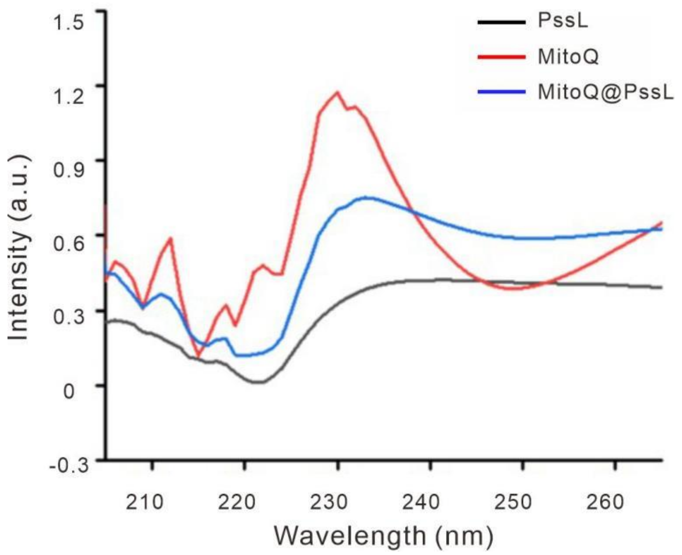

Figure S1. UV-vis absorbance spectra of MitoQ@PssL NPs.

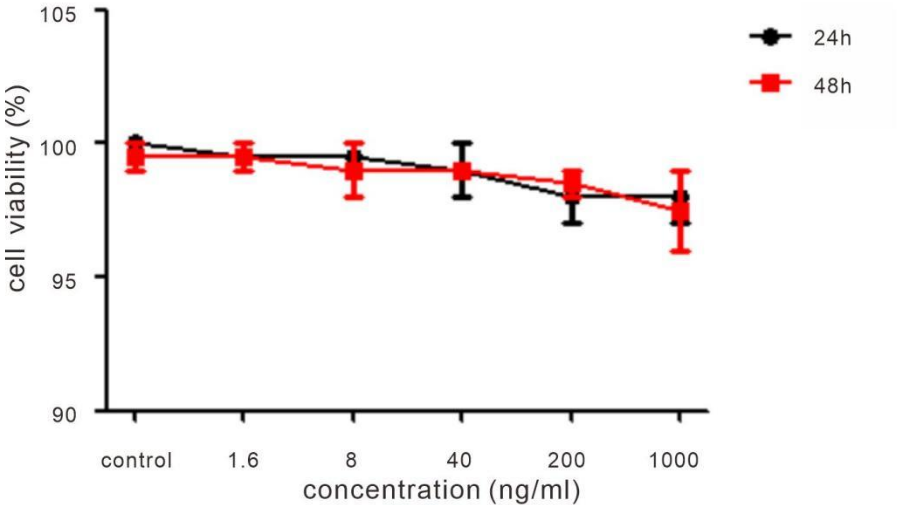

Figure S2. Cell viability after treated with MitoQ@PssL NPs for 24h and 48 h.

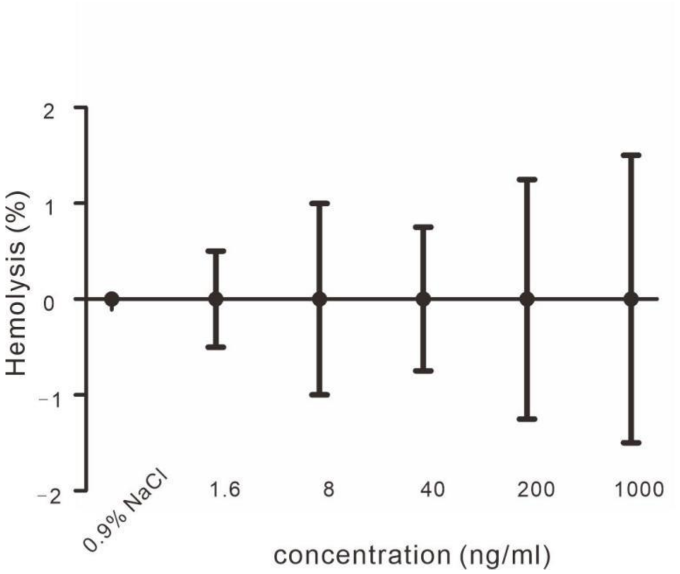

Figure S3. Hemolytic assay after treated with MitoQ@PssL NPs for 3h.

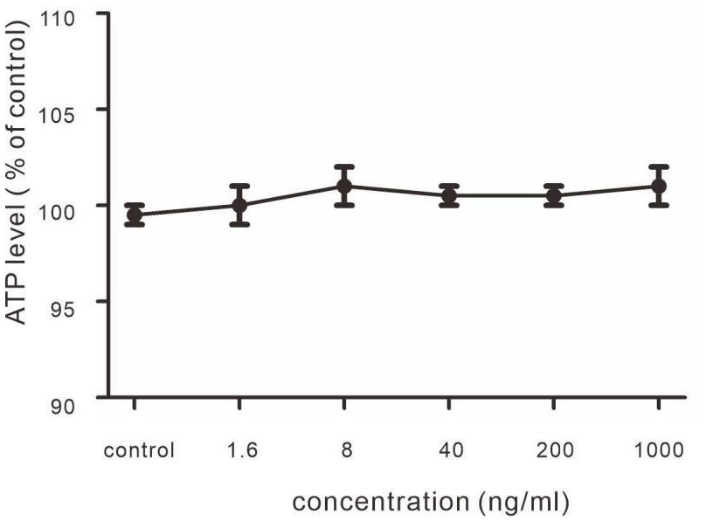

Figure S4. ATP levels after MitoQ@PssL NPs treated experiments.

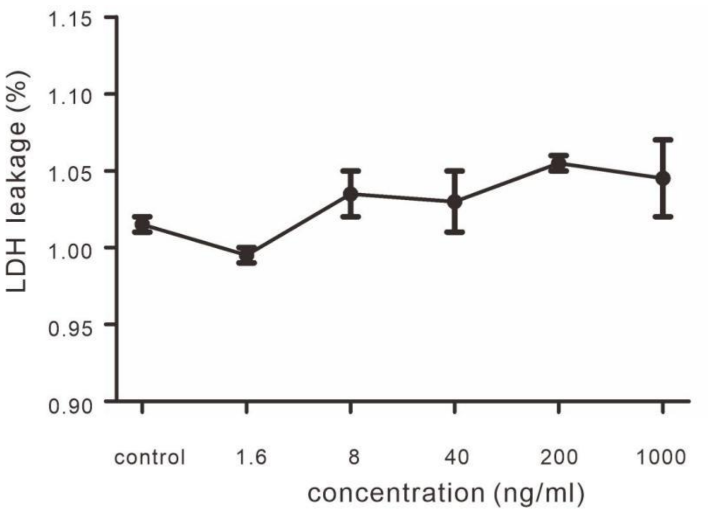

Figure S5. LDH release after MitoQ@PssL NPs treated experiments.

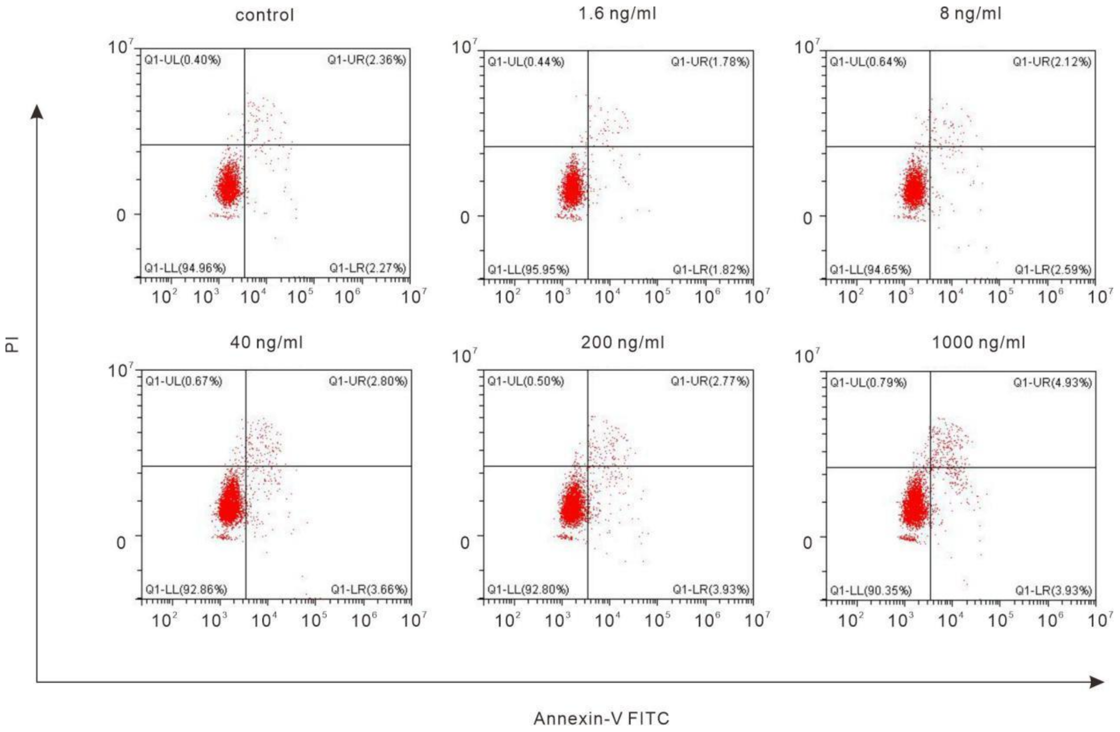

Figure S6. Apoptosis ratios detection of hPDLSCs exposed with MitoQ@PssL NPs at different concentrations.

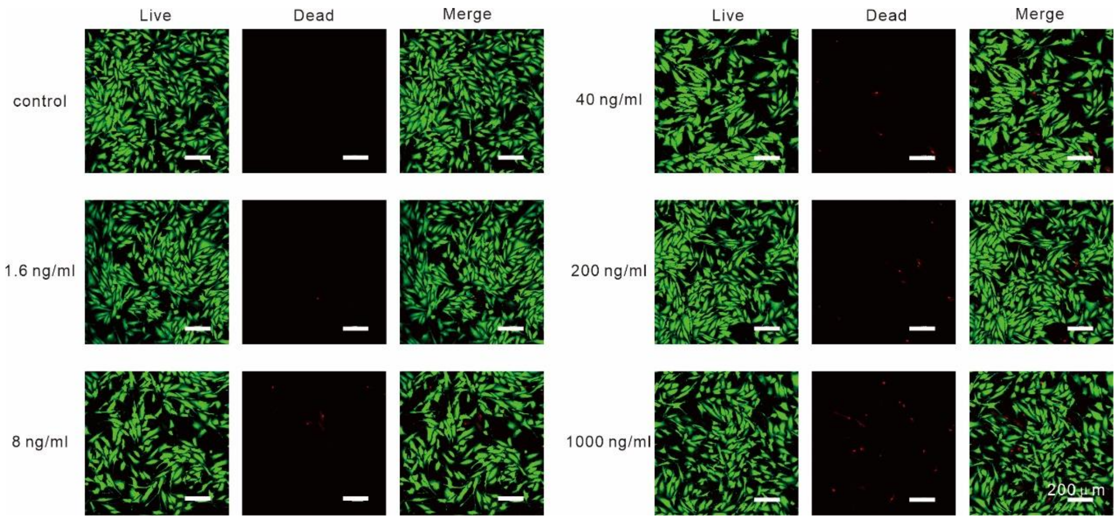

Figure S7. Live/dead dual-stained fluorescence images of cells incubated with MitoQ@PssL NPs at different concentrations.

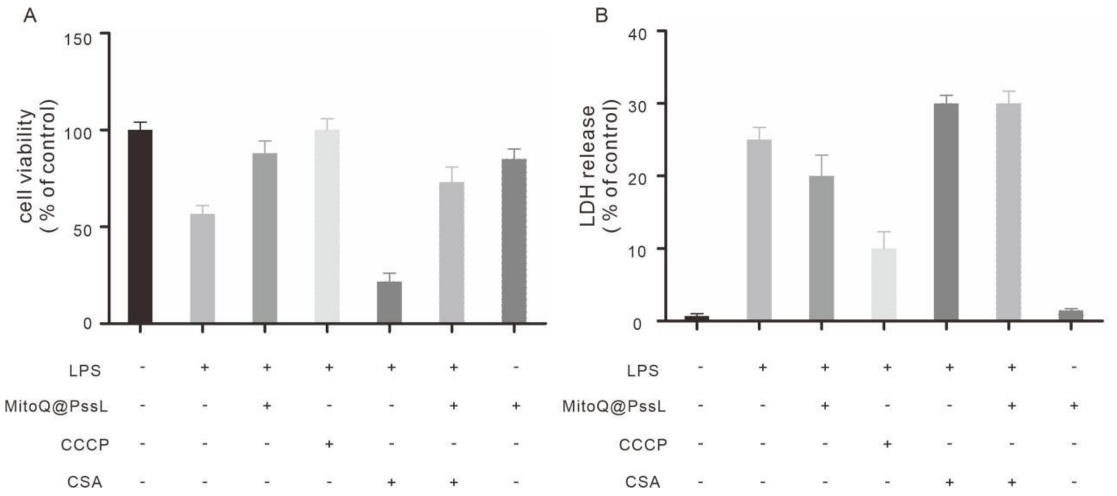

Figure S8. (A) The cell viability of hPDLSCs. (B) The release of LDH in culture medium of hPDLSCs.

Tiple asterisks indicate P<0.001.

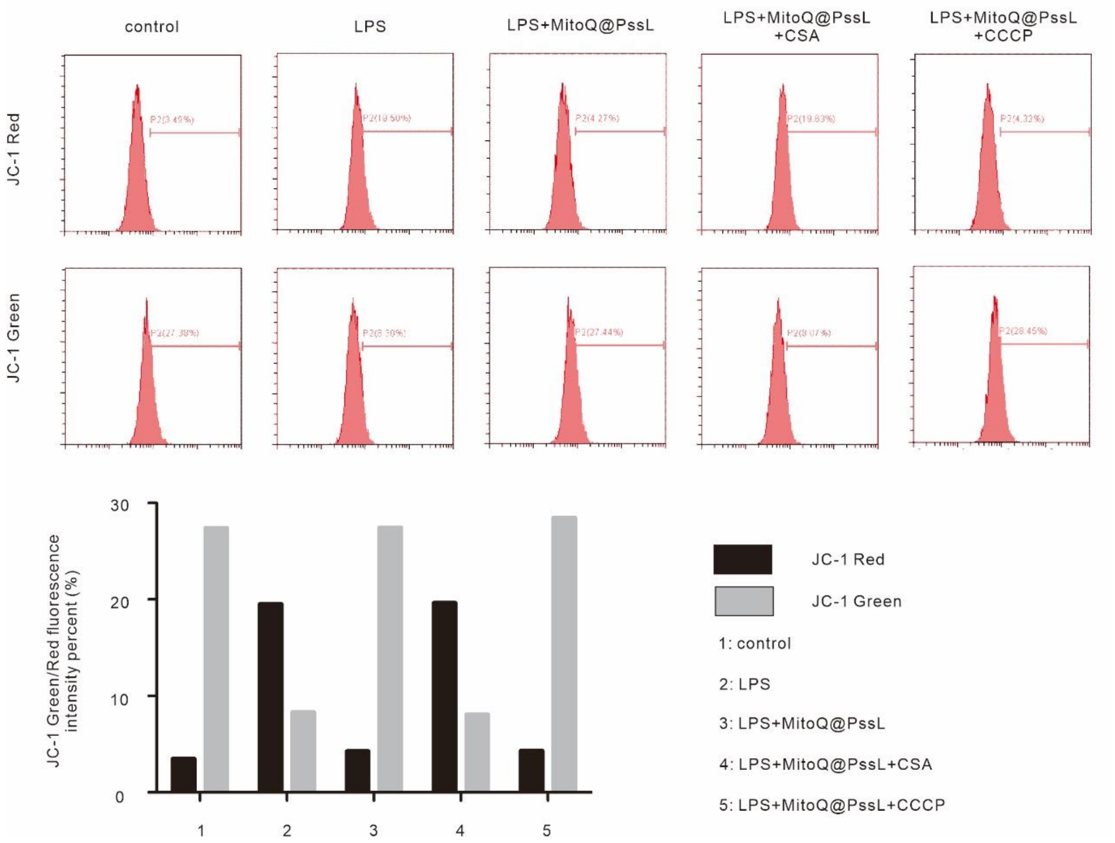

Figure S9. Mitochondrial membrane potential (ΔΨm) was assessed by probe JC-1.

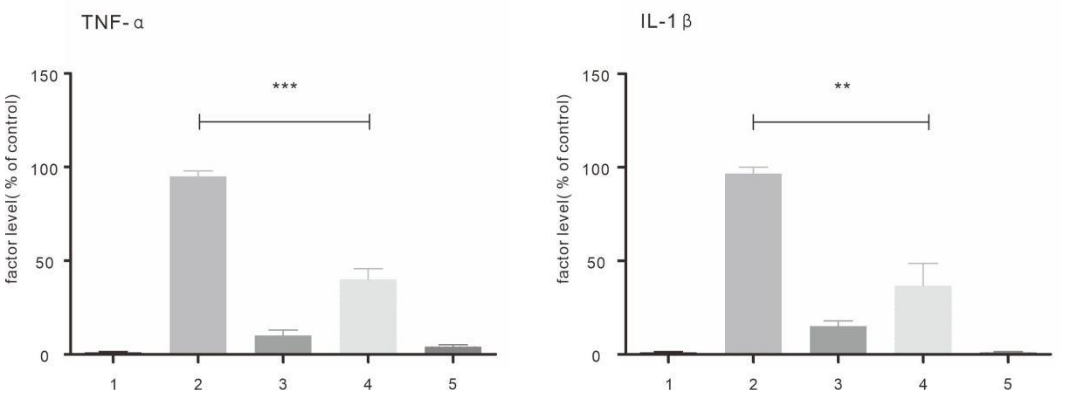

Figure S10. Expression of inflammatory mediators in LPS-treated hPDLSCs. (1:control; 2:LPS; 3:LPS+MitoQ; 4:LPS+MitoQ@PssL; 5:NAC+LPS+MitoQ@PssL) Tiple asterisks indicate P<0.001.

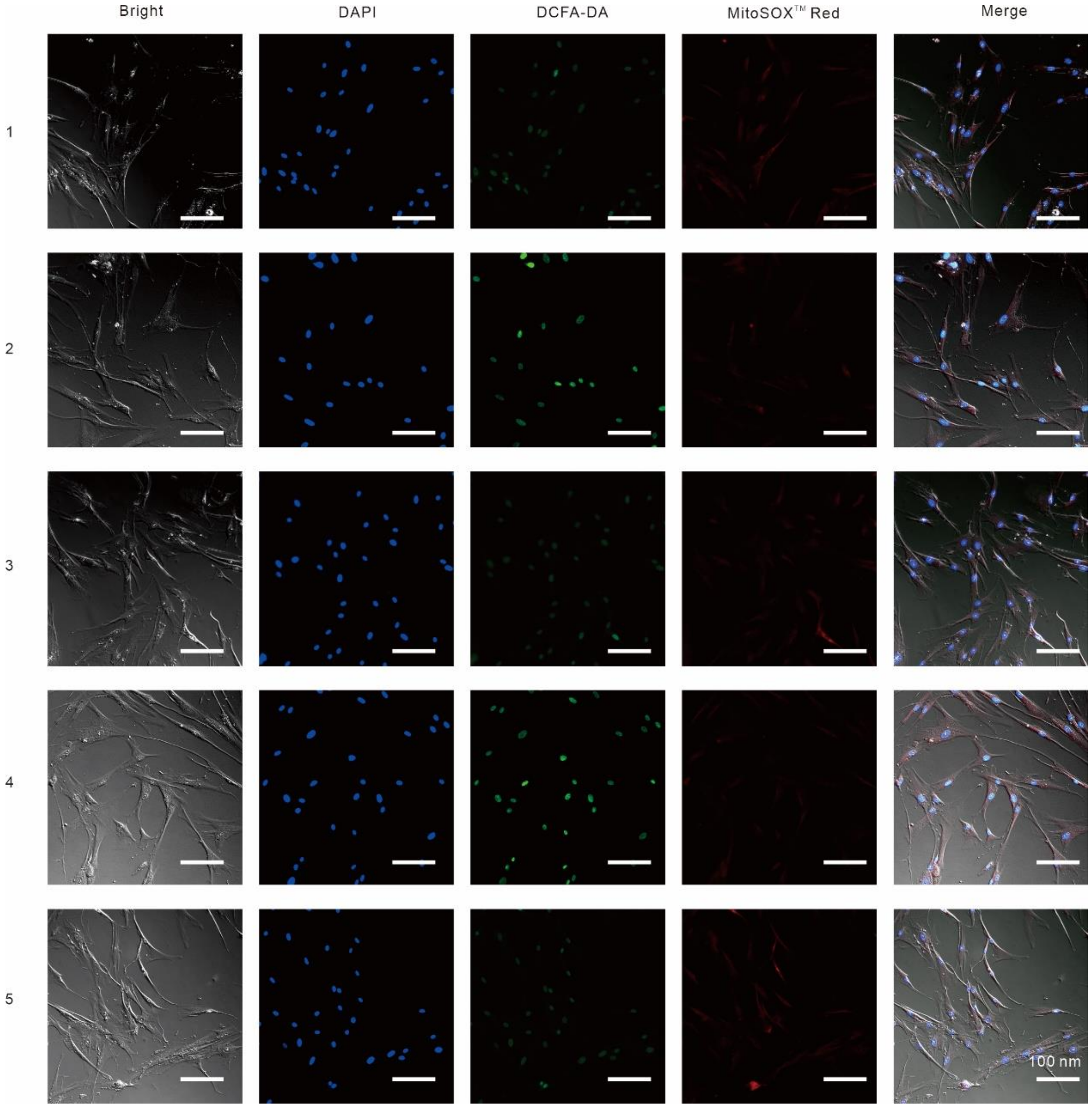

Figure S11. Fluorescence images of LPS-treated hPDLSCs.
(1:control; 2:LPS; 3:LPS+MitoQ; 4:LPS+MitoQ@PssL; 5:NAC+LPS+MitoQ@PssL)
Scale bar was equal to 100 μm.

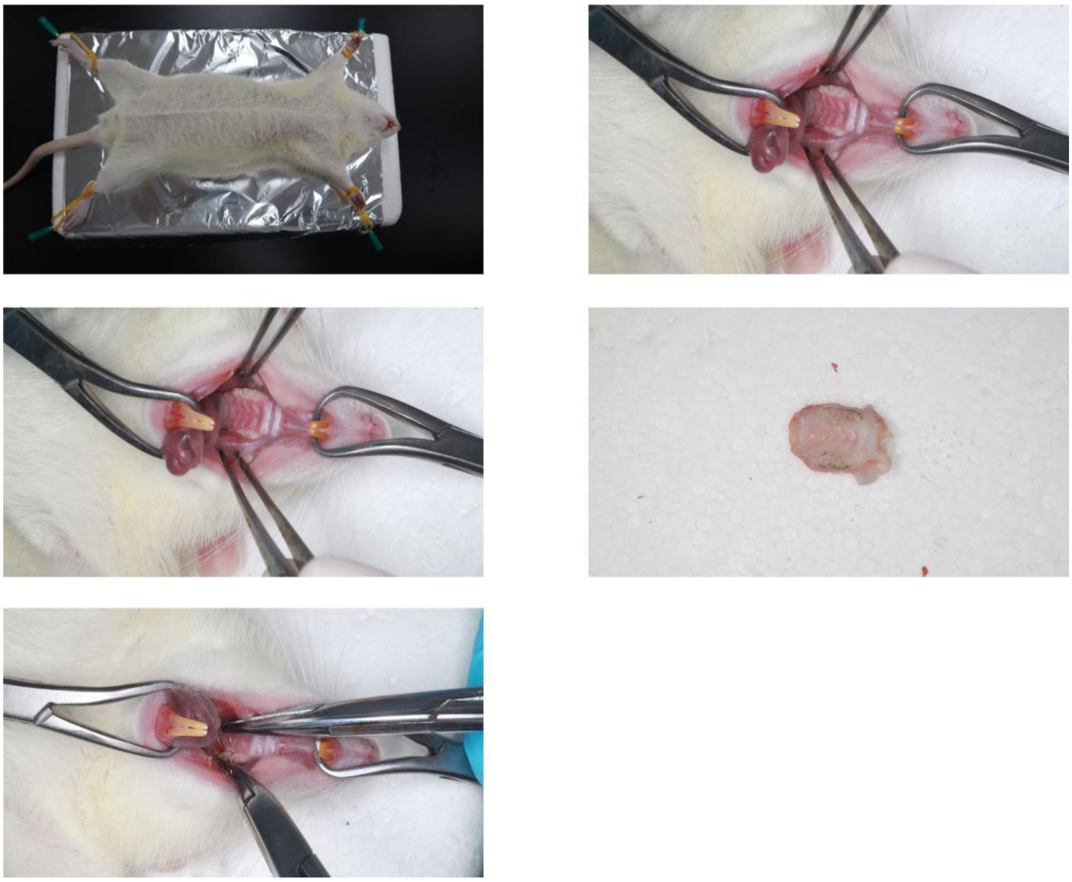

Figure S12. Surgery process of rat experimental periodontitis model.

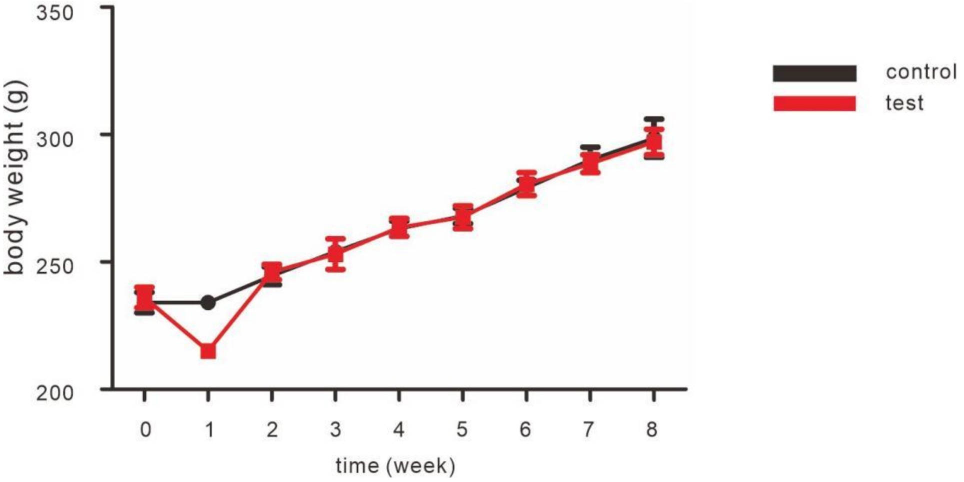

Figure S13. Rats body weights.

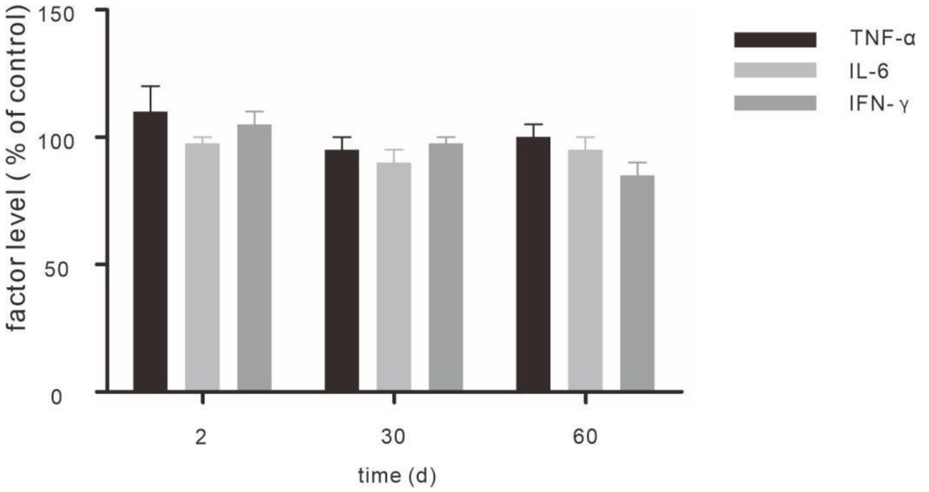

Figure S14. Cytokine response in rats.
2d（Tiple asterisks indicate P<0.05）;
30d（Tiple asterisks indicate P＞0.05）;
60d（Tiple asterisks indicate P＞0.05）.

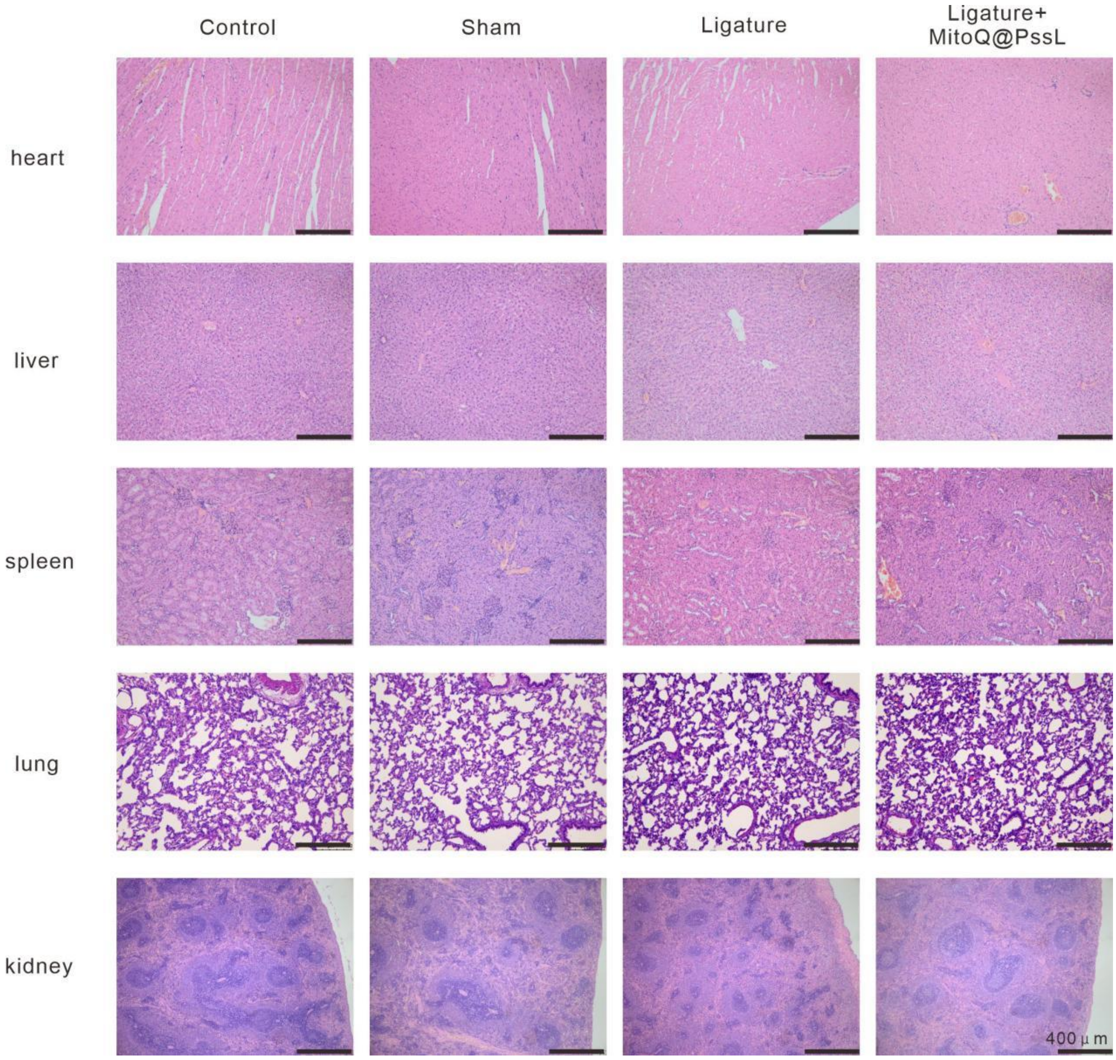

Figure S15. Histological images of main organs post-treatment.
